# Cardiorespiratory Optimisation By Arteriovenous fistula Ligation after renal Transplantation (COBALT): study protocol for a multicentre randomised interventional feasibility trial

**DOI:** 10.1136/bmjopen-2022-067668

**Published:** 2023-02-09

**Authors:** Veena Surendrakumar, Emma Aitken, Patrick Mark, Reza Motallebzadeh, James Hunter, Aimen Amer, Dominic Summers, Kirsten Rennie, Leila Rooshenas, Madalina Garbi, Karl Sylvester, Cara Hudson, Jennifer Banks, Anna Sidders, Andrew Norton, Matthew Slater, Matthew Bartlett, Simon Knight, Gavin Pettigrew

**Affiliations:** 1 Department of Thoracic Surgery, University Hospitals Birmingham NHS Foundation Trust, Birmingham, UK; 2 Department of Surgery, University of Cambridge, Cambridge, UK; 3 Department of Renal Surgery, Queen Elizabeth University Hospital, Glasgow, UK; 4 Institute of Cardiovascular and Medical Sciences, University of Glasgow, Glasgow, UK; 5 Department of Nephrology and Transplantation, Royal Free London NHS Foundation Trust, London, UK; 6 Division of Surgery and Interventional Science, University College London, London, UK; 7 Department of Transplant and Dialysis Access Surgery, University Hospitals Coventry and Warwickshire NHS Trust, Coventry, UK; 8 Nuffield Department of Surgical Sciences, University of Oxford, Oxford, UK; 9 Institute of Transplantation, Newcastle Upon Tyne Hospitals NHS Foundation Trust, Newcastle Upon Tyne, UK; 10 Department of Transplant Surgery, Cambridge University Hospitals NHS Foundation Trust, Cambridge, UK; 11 MRC Epidemiology Unit, University of Cambridge, Cambridge, UK; 12 Bristol Population Health Science Institute, University of Bristol, Bristol, UK; 13 Department of Cardiology, Royal Papworth Hospital NHS Foundation Trust, Cambridge, UK; 14 Respiratory Physiology Services, Cambridge University Hospitals NHS Foundation Trust, Cambridge, UK; 15 Statistics and Clinical Studies, NHS Blood and Transplant Organ Donation and Transplantation Directorate, Bristol, UK; 16 Clinical Trials Unit, NHSBT Clinical Trials Unit, Cambridge, UK; 17 Addenbrooke's Kidney Patients Association, Cambridge, UK; 18 Vascular Studies, Royal Free London NHS Foundation Trust, London, UK

**Keywords:** renal transplantation, transplant surgery, transplant medicine

## Abstract

**Introduction:**

Cardiovascular events are a major cause of mortality following successful kidney transplantation.

Arteriovenous fistulas (AVFs) are considered the best option for haemodialysis, but may contribute to this excess mortality because they promote adverse cardiac remodelling and ventricular hypertrophy. This raises the question whether recipients with a well-functioning kidney transplant should undergo elective AVF ligation.

**Methods and analysis:**

The COBALT feasibility study is a multicentre interventional randomised controlled trial (RCT) that will randomise renal transplant patients with stable graft function and a working AVF on a 1:1 basis to standard care (continued conservative management) or to AVF ligation. All patients will perform cardiopulmonary exercise testing (CPET) on recruitment and 6 months later. Daily functioning and quality of life will be additionally assessed by questionnaire completion and objective measure of physical activity. The primary outcome—the proportion of approached patients who complete the study (incorporating rates of consent, receipt of allocated intervention and completion of both CPETs without withdrawal)—will determine progression to a full-scale RCT. Design of the proposed RCT will be informed by an embedded qualitative assessment of participant and healthcare professional involvement.

**Ethics and dissemination:**

This study has been approved by the East Midlands—Derby Research Ethics Committee (22/EM/0002) and the Health Research Authority. The results of this work will be disseminated academically through presentation at national and international renal meetings and via open access, peer-reviewed outputs. Existing networks of renal patient groups will also be used to disseminate the study findings to other key stakeholders.

**Trial registration number:**

ISRCTN49033491.

**STRENGTHS AND LIMITATIONS OF THIS STUDY**

- This study will undertake a gold-standard assessment of cardiorespiratory fitness following arteriovenous fistula (AVF) ligation, with the correlate to daily activity levels provided by continuous monitoring via wearable devices.
- The inclusion of quality of life evaluation will ensure the trial is patient centred, with its outcomes immediately relevant to transplant recipients.
- The qualitative component will involve patient participants as well as assess barriers to recruitment among healthcare professionals.
- This feasibility study is a cost-effective and pragmatic approach to demonstrating that the proposed full-scale randomised controlled trial (RCT) is achievable and will likely inform further modifications of the RCT design to ensure its successful delivery.
- The relatively small numbers required for the feasibility study will limit the ability to identify a threshold value for fistula flow rate that discriminates ‘high-flow’ from ‘low-flow’ states.

## Introduction

For most people with kidney failure, transplantation is the best form of treatment, and offers clear survival, quality of life (QoL) and cost benefits over the alternative option of continued dialysis.^1–4^ Reflecting this, there are now 41 000 people in the UK with a working kidney transplant, which is more than are currently receiving dialysis.^5^ Nevertheless, when compared with the age-matched general population, life expectancy with a working transplant remains poorer,^6 7^ with the incidence of cardiovascular disease in kidney transplant patients almost five times greater than the age-matched general population.^8^ Consequently, recipient death with a functioning graft is now the most common cause of graft loss.^9^ The underlying conditions responsible for renal disease (most notably diabetes or hypertension) have well-documented cardiac sequelae, and additional transplant-specific factors (such as the metabolic consequences of immunosuppression) also increase cardiovascular risk. However, arteriovenous fistulas (AVFs) that are created for dialysis access prior to transplantation may also have profound, and long-lasting, haemodynamic consequences, and thus may contribute to long-term cardiac mortality in kidney transplant recipients.

AVFs are the optimal vascular access for providing haemodialysis for patients with end-stage renal disease (ESRD). Compared with the alternatives; either a central venous catheter or a synthetic arteriovenous graft; AVFs last longer and are associated with improved QoL^10 11^ and a lower incidence of sepsis-related deaths.^12 13^ UK Renal Association guidelines accordingly recommend AVFs as first choice for haemodialysis access.^14^

Nevertheless, AVFs have profound haemodynamic consequences. As an AVF ‘matures’ and the vein enlarges, the blood flow through it increases tremendously, and flows of 2–3 litres/minute are not uncommon.^15^ The drop in systemic vascular resistance triggers neurohormonal responses that increase cardiac contractility and circulating volume, leading to increased ventricular preload, stroke volume and cardiac output. This results in left ventricular (LV) hypertrophy and dilatation, and in severe cases may cause high-output cardiac failure.^16–18^ Even for relatively fit individuals, the cardiac remodelling that occurs following AVF formation likely increases their cardiovascular risk.^19 20^

There is increasing evidence that AVF disconnection improves cardiac structure in stable renal transplant recipients.^21^ It is however not yet clear if doing so will reduce their cardiovascular disease burden. The only randomised controlled trial (RCT) to date^22^ examines the impact of AVF ligation on LV mass using cardiac MRI, and although noteworthy, has its limitations as LV mass is not necessarily reflective of functional capacity, and it has as yet not engendered a change in clinical practice. This is also reflected in the attitudes of transplant patients and their clinicians who often hold strong views about whether their AVF should be preserved or ligated. Stronger evidence is therefore required to guide patient choice and to determine whether fistula disconnection should become standard care following transplantation.

Cardiorespiratory fitness (as objectively measured by cardiopulmonary exercise testing (CPET)) provides a more accurate assessment of functional capacity than imaging studies such as cardiac MRI,^23^ and is a strong independent predictor of both cardiovascular risk and all-cause mortality in various patient groups, including those with ESRD.^24–29^ Peak oxygen uptake (VO_2_, the principal CPET index) and its percentage predicted for age, sex and weight, is the gold standard to objectively assess functional limitations, with small improvements in peak VO_2_ conferring a dramatic reduction in risk of cardiovascular death.^30^ Importantly, QoL correlates with peak VO_2_ for a variety of cardiac diseases,^31–33^ and for the individual, increases in peak VO_2_ improve functional ability and QoL.^34–36^

By combining rigorous assessment of cardiorespiratory fitness with activity monitoring and QoL assessment, we aim to objectively detail how AVF disconnection changes the individual’s physical capacity and assess its impact on their general well-being. The feasibility study detailed in this protocol represents the first step in achieving this goal.

## Objectives

1. To conduct a feasibility study involving six centres, that mirrors a proposed full-scale RCT, with predefined cut-offs with regards patient recruitment and retention rates to justify progression to the larger RCT.
2. To understand patients’ and healthcare professionals’ perceived acceptability of the proposed trial design and processes.
3. To assess feasibility and acceptability of CPET in the kidney transplant population, as judged by the proportion of participants who successfully complete both tests.
4. To assess patient compliance with wearing a wrist-worn accelerometer (physical activity measurement).

Our longer term objective is to use the findings regarding recruitment and retention from this feasibility study to inform the design and powering of a future multicentre RCT that will test the hypothesis:

- In stable renal transplant patients, fistula disconnection improves cardiorespiratory fitness, thereby increasing patients’ activity levels and improving QoL.

## Methods and analysis

### Study design

**Figure 1:**
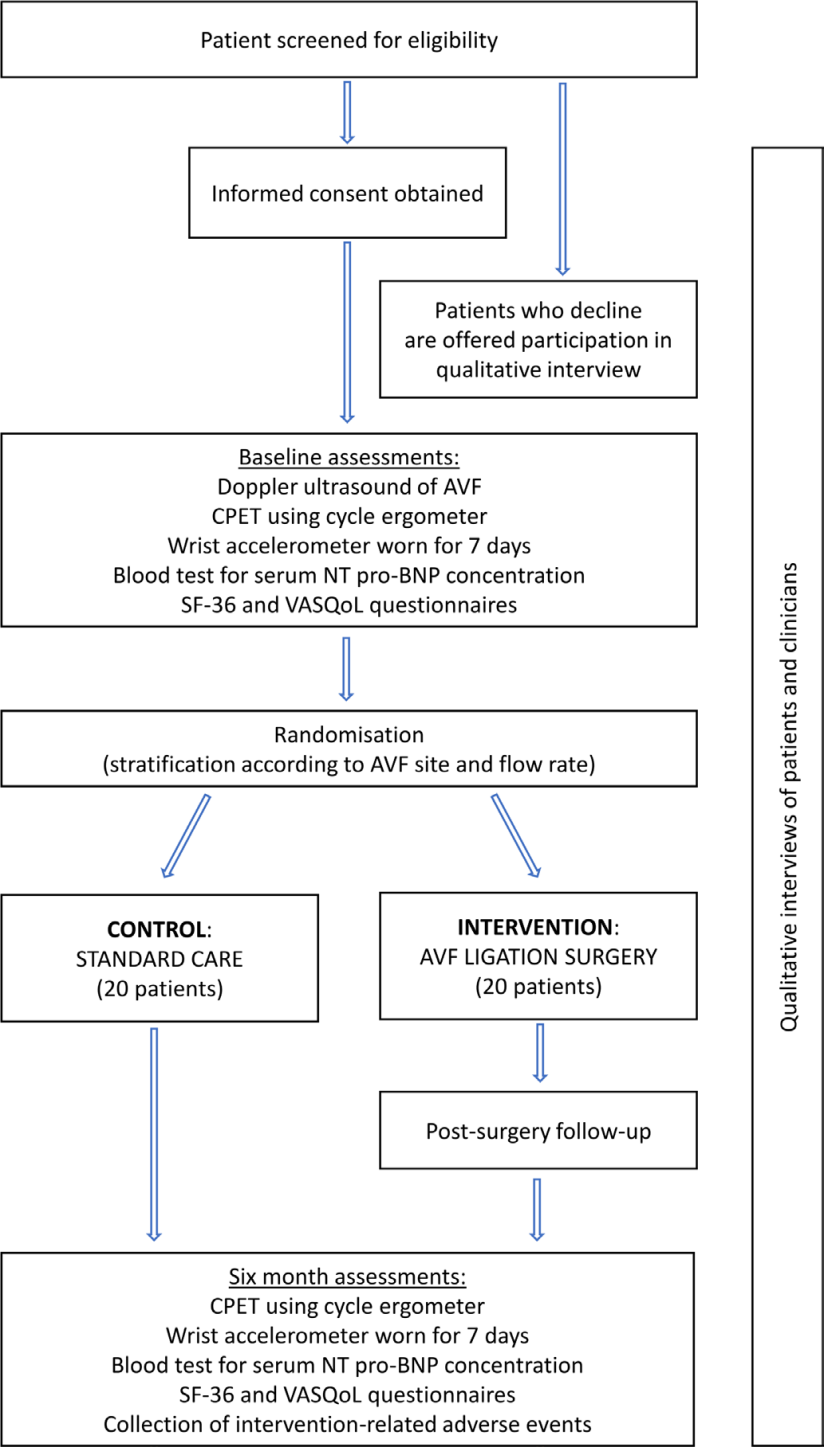
Overall design of Cardiorespiratory Optimisation By Arteriovenous fistula Ligation after renal Transplantation study. AVF, arteriovenous fistula; CPET, cardiopulmonary exercise test; NT pro-BNP, N-terminal pro-B-type natriuretic peptide; SF-36, Short Form 36; VASQoL, Vascular Access Specific Quality of Life.

This interventional, multicentre feasibility study will randomise 40 patients in a 1:1 ratio to the intervention (fistula ligation) or to continued standard care (observance of fistula, with intervention only when clinically indicated), stratified by the fistula site (wrist or elbow) and the binary variable fistula flow rate (high or low). The overall study design is outlined in figure 1.

### Study setting

The study will take place in six NHS hospitals with a vascular access service in England and Scotland: Addenbrooke’s Hospital, Cambridge; Royal Free Hospital, London; Queen Elizabeth University Hospital, Glasgow; John Radcliffe Hospital & Churchill Hospitals, Oxford; University Hospital, Coventry; Freeman Hospital, Newcastle. The study will run from 1 September 2021 until 31 July 2023, with recruitment taking place between 1 March 2022 until 30 November 2022.

### Sample selection

Participants will be considered eligible for enrolment if they fulfil the inclusion and exclusion criteria detailed below. Eligible transplant recipients will be identified by the local clinical team and approached either at their routine transplant follow-up clinic appointment or by post, with full details of the trial procedures and assessments provided at the initial screening visit. At this stage, consenting patients, as well as a small number who decline to take part in the trial, will also be invited to additionally participate in qualitative interviews exploring barriers to recruitment; this will not be a mandatory requirement for trial participation. Identifying and approaching healthcare professionals to participate in qualitative interviews will be the responsibility of the qualitative research team, drawing on their own observations from site visits and communications with the research team. Informed written consent will be obtained from all trial participants (online supplemental files).

10.1136/bmjopen-2022-067668.supp1 Supplementary data

10.1136/bmjopen-2022-067668.supp2 Supplementary data

### Participant eligibility

#### Inclusion criteria

1. Kidney transplant recipient aged 16 years or older.
2. At least 1 year from kidney transplantation and with a patent AVF.
3. Stable graft function (eGFR>35 mL/min/1.73 m^2^ (calculated by the Chronic Kidney Disease Epidemiology Collaboration (CKD-EPI)estimation^37^) and without recent rejection episode or recent decline in graft function).
4. Adequate English to understand the study information by verbal explanation and the written participant information sheet.
5. Capacity to provide full informed consent.

#### Exclusion criteria

1. Patients lacking capacity.
2. Those in whom CPET is contraindicated, as per national guidance
3. Those considered to have no further first-line options for AVF creation at their wrist or elbow, as assessed by a vascular access specialist.
4. Patients in whom AVF disconnection is indicated on clinical grounds.

### Intervention

This trial will have one standard care control arm and one intervention arm, as described below.

#### Control: standard care

This will involve continued conservative management, with fistula disconnection only performed if clinically indicated.

#### Intervention: fistula disconnection surgery

This comprises either surgical division and oversewing of the fistula vein at the site of the original anastomosis on the artery, or disconnection of the fistula vein entirely from the artery at the anastomotic site. The operation may also include excision of the venous outflow segment if particularly enlarged or aneurysmal. The procedure may be performed under local, regional, or general anaesthesia, typically as a day case.

### Outcomes

For this feasibility study, the primary outcome measure will be the proportion of approached patients who complete the study, defined as the proportion of eligible approached patients that consent, receive the allocated intervention, and complete both CPET tests without withdrawing.

Secondary outcomes are subdivided into: those related to the feasibility of the study; clinical measures that will be measured at baseline and 6 months following intervention, and the differences in these values; and safety outcome measures occurring between study consent and the 6-month assessment visit.

#### Feasibility outcome measures

1. Proportion of approached patients who consent to participation.
2. Proportion of participants who receive the allocated intervention.
3. Proportion of participants who complete both CPETs.
4. Proportion of participants who did not withdraw.
5. Proportion of participants who received the allocated intervention and completed both CPETs without withdrawing.
6. Proportion of participants who were compliant with wearing wrist accelerometers.
7. Rate of patient recruitment at each trial centre.
8. Time (days) from providing consent to first CPET and from CPET to fistula disconnection.
9. Patient and healthcare professional perceived acceptability of trial design and processes (qualitative assessment carried out by interviews).

#### Clinical outcome measures

1. Peak VO_2_ as measured during incremental CPET on a cycle ergometer.
2. Physical functioning domain score of Short Form-36 Quality of Life questionnaire.^38^
3. Activity levels (mean daily Euclidean norm minus one) as calculated from accelerometry data collected from provided wrist-worn accelerometer device over 7-day period at baseline and following 6-month assessment.
4. Serum N-terminal pro B-type natriuretic peptide value.
5. Office blood pressure.
6. Additional CPET indices: ventilatory anaerobic threshold, endurance time, peak workload, O_2_ pulse, VO_2_/work rate slope (VO_2_/WR), minute ventilation/CO_2_ output slope (VE/VCO_2_), heart rate/VO_2_ slope (HR/VO_2_), perceived exertion rating (Borg scale).
7. Additional domain scores of the Short Form-36 QoL questionnaire^38^: physical role limitation, emotional role limitation, energy/ fatigue, emotional well-being, social functioning, pain, general health, perceived health change.
8. Kidney transplant function (eGFR by CKD-EPI estimation^37^).
9. Fistula-related symptoms reported in modified Vascular Access Specific QoL questionnaire^39^ (for all participants at enrolment, and at 6 months for cohort randomised to standard care).
10. Proportion of participants with at least one major adverse cardiovascular event (MACE)^40^: cardiovascular death; ischaemic cardiovascular event (myocardial infarction, percutaneous coronary intervention, coronary artery bypass graft); hospitalisation for heart failure at any time during the 6-month study period.

#### Safety outcome measures

1. Surgical complications from fistula disconnection (number in each Clavien-Dindo classification^41^).
2. Fistula-related complications in the standard care group.
3. Transplant failure (return to permanent dialysis or retransplantation).
4. Hospitalisation (number of hospitalisations and cumulative days in hospital).
5. Patient death (all cause).

### Qualitative substudy

We will integrate qualitative research throughout the feasibility study, with the overall aim of identifying and understanding issues that might threaten the viability of a full-scale future RCT. This includes issues of recruitment and informed consent, and acceptability of the interventions and proposed outcome measure assessments.

The specific objectives of the integrated qualitative research are to:

1. investigate healthcare professionals’ perspectives on the trial design, including perceptions of equipoise, views on the eligibility criteria, relevance of the primary and secondary outcomes, and approaches to measuring these.
2. understand the acceptability of randomisation from patients’ perspectives, with a focus on reasons for accepting/declining trial participation and factors that influenced these decisions.
3. explore patients’ experiences of trial participation, including perceived acceptability of being in the intervention/control arms and undergoing outcome assessments (eg, CPET, wearing accelerometers).

We will address the above objectives by conducting semistructured interviews with healthcare professionals and patients who accept and decline trial participation. Both healthcare professionals and patients will be sampled across all six sites. We anticipate up to 40 interviews will be conducted (20 patients, 20 clinical professionals) over the 2-year study period.

### Progression to full-scale RCT

This feasibility study aims to test the processes of a proposed future multicentre RCT and therefore we will use a traffic-light approach to determine trial progression according to the following indices:

- Patient participation:
  - Green: > 40% of approached patients consent to trial participation.
  - Amber: Between 30% and 40% of approached patients consent.
  - Red: < 30% of patients consent.
- Adherence to randomisation and study completion:
  - Green: > 85% of enrolled participants received the allocated intervention and completed both CPETs without withdrawing.
  - Amber: Between 75% and 85% completion rate.
  - Red: < 75% completion rate.

We anticipate that a green light for the above indices will realise patient recruitment and retention rates within a future full-scale RCT to enable scheduled completion. In response to an amber trigger, the Trial Management Group (TMG) will develop strategies that specifically respond to potential issues with patient participation that have been highlighted by the qualitative analysis. We would not proceed to a full-scale trial if a red signal is triggered.

During this feasibility study, we expect that, once open, each of the participating centres recruits a minimum of one patient per month and that fistula disconnection is performed within 62 days of CPET. If not met, the TMG will attempt to identify and address systematic problems that may hinder the main RCT.

### Sample size

As this is a feasibility study, formal sample size calculation is not required. We will therefore aim to recruit 40 patients (in keeping with recommendations^42^), 20 in each arm, with progression to a future RCT dependent on meeting set criteria relating to participant recruitment and retention rates as above. If the percentage of patients completing the study is 85%, 40 patients will give a 95% CI (70.2 to 94.3).

### Randomisation

Randomisation of consenting participants will be stratified according to fistula site (wrist or elbow) and fistula volume flow (low or high), as determined by a pre-randomisation Doppler ultrasound of the fistula, and allocated in a 1:1 ratio to the intervention (surgical disconnection of fistula) or to standard care (continued conservative management) using an interactive web response system provided by Sealed Envelope. Fistula flow rate will be measured during baseline assessment visits and categorised as either low flow (wrist AVF: ≤800 mL/min, elbow AVF: ≤1400 mL/min) or high flow (wrist AVF: >800 mL/min, elbow AVF: >1400 mL/min). The research team will confirm to the clinical and surgical teams which arm of the trial the participant has been randomised to, with the intervention being performed by a vascular access/transplant surgeon within 62 days of completion of baseline assessments. Given the surgical nature of the intervention, there will be no blinding in this trial with all participants, clinical and research team members aware of which trial arm is assigned at randomisation.

### Statistical methods

The analysis of the primary outcome of the proportion of approached patients who complete the study successfully will include the proportion of eligible approached patients who consented, received the allocated intervention, completed both CPET tests and did not withdraw, with 95% CIs. As part of the intention-to-treat approach, those who have graft failure, die, or are lost to follow-up will be included in the analysis of the primary outcome as not completing the study successfully. The component proportions and the corresponding 95% CIs of the primary outcome will be presented as secondary outcomes.

The secondary feasibility outcomes of recruitment rate by trial site and the proportion of participants who were compliant with wearing wrist accelerometers, and their 95% CIs, will also be presented. Compliance to wearing the monitor will be assessed by measuring wear time. The days from providing consent to first CPET and the days from CPET to fistula disconnection will be summarised, and the median and IQR presented. Patient and healthcare professional perceived acceptability of the trial design and processes will be analysed using qualitative analysis.

Descriptive statistics of the secondary clinical outcomes will be presented for each arm separately, at baseline, the 6-month assessment, and the average change over time, with 95% CIs. Peak VO_2_ will also be presented by fistula site and according to prespecified flow rate categories. Very limited hypothesis testing will be conducted but mixed linear regression will be used to examine any difference in peak VO_2_ at 6 months between the two treatment arms, with adjustment for fistula site, flow rate, baseline peak VO_2_ and centre. Fistula-related symptoms will be assessed using the modified VASQoL questionnaire for all participants at enrolment and at 6 months for the standard care arm and compared where applicable. The proportion of participants who experienced at least one MACE by 6 months will be presented (alongside 95% CIs), and details of all reported MACEs listed. In addition, summaries of all secondary safety outcomes will be presented by arm.

## Patient and public involvement

The views of both kidney transplant patients and members of the public were sought during the design of this study, both through Patient and Public Advisory Group (PPAG) meetings and distributed surveys, with final oversight from local kidney patient groups. Key aspects of the study protocol were developed following these discussions with both adaptation to the study inclusion criteria and greater emphasis placed on the incorporation of QoL assessment and daily functioning.

The feasibility study has been designed such that continued patient involvement will be a core aspect, with the integrated qualitative research aimed at capturing the participant experience and generating insight into the factors that may undermine recruitment and threaten the viability of a full-scale RCT.

We have included a patient representative as part of the TMG and will also conduct 6 monthly PPI meetings during the feasibility study, with lay-person involvement, to moderate discussions with patient and clinicians on their views of trial participation.

We have already established close links with local kidney patient groups at each of the trial centres; the qualitative analysis will be shared with these groups; and we anticipate that the ensuing discussions with both patients and public will promote awareness of the study, facilitate patient recruitment and retention as well as support the dissemination of results and design of the subsequent RCT.

## Ethics and dissemination

This study has been approved by the East Midlands—Derby Research Ethics Committee (22/EM/0002) and the Health Research Authority. Research will be conducted in compliance with the approved protocol, the Declaration of Helsinki (2013), the Principles of Good Clinical Practice, the UK Data Protection Act, the UK Framework for Health and Social Care Research and any other applicable national regulations. Written informed consent will be obtained from the participants by a suitably qualified member of the clinical/research team who has received trial training. The patient’s right to refuse participation in the trial and withdraw from the study at any time and without reason will be respected, without prejudice to their further treatment.

The results of this trial will be presented to academic and non-academic groups with PPAG involvement in disseminating the study findings into the public domain. Open access, peer-reviewed academic outputs and research reports together with associated summaries and key findings will be produced and held on the study website.

## Access to data

Access to the final dataset will be available on request from Prof Gavin Pettigrew, gjp25@cam.ac.uk, after the trial results have been published (expected September 2024) and should continue to be available for 4 years. No identifiable data will be shared. All participants have provided their written informed consent for their data to be used in this way.

## Discussion

There is no clear guideline on the management of AVFs following kidney transplantation. However, with the known burden of cardiovascular disease among renal transplant recipients, and the existing evidence that AVF disconnection improves cardiac structure, there is an increasing need to establish the role of AVF ligation in improving cardiovascular outcomes. Given the varying opinions of transplant patients and their clinicians on AVF preservation versus ligation, the COBALT feasibility study is a necessary requirement prior to pursuing a definitive robust RCT. With its embedded qualitative component, this feasibility trial aims to gauge the acceptability of the proposed trial design and optimise patient recruitment, with predefined stop–go criteria to justify progression to a full-scale RCT.

## Supplementary Material

Author's manuscript
