# Supplementary material for: Cardiorespiratory Optimisation By Arteriovenous fistula Ligation after renal Transplantation (COBALT): study protocol for a multicentre randomised interventional feasibility trial
Source: BMJ Open. 2023 Feb 9;13(2):e067668. doi: 10.1136/bmjopen-2022-067668 (PMC9923321; doi:10.1136/bmjopen-2022-067668)
Supplement: Supplementary data [file bmjopen-2022-067668supp001.pdf]

COBALT

**Participant Identification Number:** ______________

University of BRISTOL

TRUST LOGO

# Informed Consent Form for Healthcare Professionals

Should we ligate haemodialysis fistulas in patients after they have been transplanted successfully: the **COBALT** feasibility study (**C**ardiorespiratory **O**ptimisation **B**y **A**VF **L**igation after **T**ransplantation).

**Please initial the boxes if you agree with the following statements:**

1. I confirm that I have read the Healthcare Professionals information sheet dated.................. (version...........) for the above study. I have had the opportunity to consider the information, ask questions and have had these answered satisfactorily. ☐
2. I agree to taking part in an audio-recorded interview. ☐
3. I understand that I am free to withdraw from the interview study at any time, without giving a reason, and that withdrawing will not affect my legal rights. I understand that withdrawal of data already provided will not be possible after 2 weeks of recordings having taken place. ☐
4. I agree to take part in the above study. ☐

**Optional statements (please initial either the yes or no box):**

| Statement | Yes | No |
|---|---|---|
| 5. I am happy to be contacted in the future for a potential follow-up interview, and understand I have a right to accept/decline taking part at the time. | ☐ | ☐ |
| 6. I agree to my audio-recorded interviews being transferred to and retained by the University of Bristol and their authorised representatives for transcription, training, teaching and research purposes, now and in the future. | ☐ | ☐ |
| 7. I agree to anonymised data from my interviews being made “Controlled access” after the study, and understand this means data will be stored indefinitely and may be used for purposes not related to this study, although it will not be possible to identify me from these data. | ☐ | ☐ |

| ________________________ | ______________ | ____________________ |
|---|---|---|
| Name of Participant | Date | Signature |
| ____________________ | _____________ | ____________________ |
| Name of person taking consent | Date | Signature |

*When completed: 1 for participant; 1 for researcher*

COBALT HCP Informed Consent Form V2.0, 19th January 2022 IRAS:305610
