# Supplementary material for: Cardiorespiratory Optimisation By Arteriovenous fistula Ligation after renal Transplantation (COBALT): study protocol for a multicentre randomised interventional feasibility trial
Source: BMJ Open. 2023 Feb 9;13(2):e067668. doi: 10.1136/bmjopen-2022-067668 (PMC9923321; doi:10.1136/bmjopen-2022-067668)
Supplement: Supplementary data [file bmjopen-2022-067668supp002.pdf]

COBALT

Insert Trust logo

IRAS ID: 305610

Title of Study: Cardiorespiratory Optimisation By Arteriovenous fistula Ligation after Transplantation (COBALT)

Centre Number: ___________

Name of Researcher: ______________________________

Participant Identification Number for this study: __________________

INFORMED CONSENT FORM

Please initial the boxes if you agree with the following statements:

1. I confirm that I have read the information sheet dated................... (version........) for the above study and that the trial procedures and information have been explained to me. I have had the opportunity to consider the information, ask questions and have had these answered satisfactorily. ☐

2. I understand that my participation is voluntary and that I am free to withdraw at any time without giving any reason, without my medical care or legal rights being affected. ☐

3. I understand that relevant sections of my medical notes and data collected during the study, may be looked at by individuals from Cambridge University Hospitals NHS Foundation Trust / University of Cambridge / NHS Blood and Transplant, from regulatory authorities or from the NHS Trust, where it is relevant to my taking part in this research. I give permission for these individuals to have access to my records. ☐

4. I understand that the information collected about me will be used to support other ethically approved research in the future, and may be shared anonymously with other researchers in the UK or abroad. ☐

Surendrakumar V, *et al. BMJ Open* 2023; 13:e067668. doi: 10.1136/bmjopen-2022-067668

COBALT

Insert Trust logo

5. I agree to take part in the COBALT study. ☐

Optional statements (please initial either the yes or no box): **Yes** **No**

6. I agree to my General Practitioner and Nephrologist (kidney doctor) being informed of my participation in the COBALT study. ☐ ☐

7. I agree that my name and contact details can be securely shared with researchers at the University of Bristol and that those researchers can contact me to arrange an interview about my experience of being approached to take part in the COBALT study (and, my experiences of participation, if relevant). ☐ ☐

8. I agree to anonymised data from my interviews being made "Controlled access" after the study and understand this means data will be stored indefinitely and may be used for purposes not related to this study, although it will not be possible to identify me from these data. ☐ ☐

Name of Participant	Date	Signature
Name of Researcher receiving consent	Date	Signature

1 x copy for participant

1 x copy for COBALT file to be kept at hospital site

1 x copy for participant's hospital medical record

Surendrakumar V, *et al. BMJ Open* 2023; 13:e067668. doi: 10.1136/bmjopen-2022-067668
