# Supplementary material for: Cardiorespiratory Optimisation By Arteriovenous fistula Ligation after renal Transplantation (COBALT): study protocol for a multicentre randomised interventional feasibility trial
Source: BMJ Open. 2023 Feb 9;13(2):e067668. doi: 10.1136/bmjopen-2022-067668 (PMC9923321; doi:10.1136/bmjopen-2022-067668)
Supplement: Author's manuscript [file bmjopen-2022-067668.draft_revisions.pdf]

BMJ Open

BMJ Open is committed to open peer review. As part of this commitment we make the peer review history of every article we publish publicly available.

When an article is published we post the peer reviewers' comments and the authors' responses online. We also post the versions of the paper that were used during peer review. These are the versions that the peer review comments apply to.

The versions of the paper that follow are the versions that were submitted during the peer review process. They are not the versions of record or the final published versions. They should not be cited or distributed as the published version of this manuscript.

BMJ Open is an open access journal and the full, final, typeset and author-corrected version of record of the manuscript is available on our site with no access controls, subscription charges or pay-per-view fees (http://bmjopen.bmj.com).

If you have any questions on BMJ Open's open peer review process please email info.bmjopen@bmj.com

Cardiorespiratory Optimisation By Arteriovenous fistula Ligation after renal Transplantation (COBALT): study protocol for a multicentre randomised interventional feasibility trial

Journal:	*BMJ Open*
Manuscript ID	bmjopen-2022-067668
Article Type:	Protocol
Date Submitted by the Author:	22-Aug-2022
Complete List of Authors:	Surendrakumar, Veena; University Hospitals Birmingham NHS Foundation Trust<br>Aitken, Emma; Queen Elizabeth University Hospital; University of Glasgow, Institute of Cardiovascular and Medical Sciences<br>Mark, Patrick; University of Glasgow, Institute of Cardiovascular and Medical Sciences<br>Motallebzadeh, Reza; Royal Free London NHS Foundation Trust; University College London<br>Hunter, James; University Hospitals Coventry and Warwickshire NHS Trust; University of Oxford<br>Amer, Aimen; Newcastle Upon Tyne Hospitals NHS Foundation Trust<br>Summers, Dominic ; Cambridge University Hospitals NHS Foundation Trust<br>Rennie, Kirsten ; University of Cambridge, MRC Epidemiology Unit<br>Rooshenas, Leila; University of Bristol<br>Garbi, Madalina ; Royal Papworth Hospital NHS Foundation Trust<br>Sylvester, Karl; Cambridge University Hospitals NHS Foundation Trust<br>Hudson, Cara; NHS Blood and Transplant Organ Donation and Transplantation Directorate, Statistics and Clinical Studies<br>Banks, Jennifer; NHS Blood and Transplant Organ Donation and Transplantation Directorate, Statistics and Clinical Studies<br>Sidders, Anna; NHSBT Clinical Trials Unit, Clinical Trials Unit<br>Norton, Andrew; Addenbrooke's Kidney Patients Association<br>Slater, Matthew; Cambridge University Hospitals NHS Foundation Trust<br>Bartlett, Matthew; Royal Free London NHS Foundation Trust, Vascular Studies<br>Knight, Simon; University of Oxford<br>Pettigrew, Gavin ; University of Cambridge, Surgery; Cambridge University Hospitals NHS Foundation Trust<br>trial group, COBALT; NHS Blood and Transplant Organ Donation and Transplantation Directorate
Keywords:	Renal transplantation < NEPHROLOGY, Transplant surgery < SURGERY, Transplant medicine < INTERNAL MEDICINE

SCHOLARONE™
Manuscripts

Cardiorespiratory Optimisation By Arteriovenous fistula Ligation after renal Transplantation (COBALT): study protocol for a multicentre randomised interventional feasibility trial

Authors
Veena Surendrakumar
University Hospitals Birmingham NHS Foundation Trust
veena.surendrakumar@nhs.net

Emma Aitken
Queen Elizabeth University Hospital, Glasgow; Institute of Cardiovascular and Medical Sciences, University of Glasgow
emma.aitken@ggc.scot.nhs.uk

Patrick B Mark
Institute of Cardiovascular and Medical Sciences, University of Glasgow
Patrick.Mark@glasgow.ac.uk

Reza Motallebzadeh
Royal Free London NHS Foundation Trust and University College London
reza.motallebzadeh@nhs.net

James P Hunter
University of Oxford and University Hospitals Coventry and Warwickshire NHS Trust
james.hunter@nds.ox.ac.uk

Aimen Amer
Newcastle Upon Tyne Hospitals NHS Foundation Trust
aimen.amer@nhs.net

Dominic Summers
Cambridge University Hospitals NHS Foundation Trust
dms39@cam.ac.uk

Kirsten L Rennie
MRC Epidemiology Unit, University of Cambridge
Kirsten.Rennie@mrc-epid.cam.ac.uk

Leila Rooshenas
University of Bristol
leila.Rooshenas@bristol.ac.uk

Madalina Garbi
Royal Papworth Hospital NHS Foundation Trust
madalina.garbi@nhs.net

Karl Sylvester
Cambridge University Hospitals NHS Foundation Trust
karl.sylvester@nhs.net

Cara Hudson
NHS Blood and Transplant Clinical Trials Unit
Cara.Hudson@nhsbt.nhs.uk

Jenni Banks
NHS Blood and Transplant Clinical Trials Unit
jenni.banks@nhsbt.nhs.uk

For peer review only

Anna Sidders
NHS Blood and Transplant Clinical Trials Unit
Anna.Sidders@nhsbt.nhs.uk

Andrew Norton
Addenbrooke's Kidney Patients Association
andrew.j.norton@pdmassociates.co.uk

Matthew Slater
Cambridge University Hospitals NHS Foundation Trust
matthewslater@nhs.net

Matthew Bartlett
Royal Free London NHS Foundation Trust
matthew.bartlett@nhs.net

Simon Knight
University of Oxford
simon.knight@nds.ox.ac.uk

Gavin J Pettigrew
University of Cambridge
gjp25@cam.ac.uk

COBALT trial group
COBALT@nhsbt.nhs.uk

KEYWORDS
Arteriovenous fistula
Ligation
Kidney transplant
Cardiopulmonary exercise testing
Quality of life

WORD COUNT
2653 words

Corresponding Author:
Mr Gavin J Pettigrew
Department of Surgery
University of Cambridge
Box 202, Level E9 Addenbrooke's Hospital
Hills Road
Cambridge
CB2 0QQ

gjp25@cam.ac.uk

Tel: 01223 336976

For peer review only

ABSTRACT

Introduction

Cardiovascular events are a major cause of mortality following successful kidney transplantation. Arteriovenous fistulas (AVFs) are considered the best option for haemodialysis, but may contribute to this excess mortality because they promote adverse cardiac remodelling and ventricular hypertrophy. This raises the question whether recipients with a well-functioning kidney transplant should undergo elective AVF ligation.

Methods and analysis

The COBALT feasibility study is a multicentre interventional randomised controlled trial (RCT) that will randomise renal transplant patients with stable graft function and a working AVF on a 1:1 basis to standard care (continued conservative management) or to AVF ligation. All patients will perform cardiopulmonary exercise testing (CPET) on recruitment and six months later. Daily functioning and quality of life (QoL) will be additionally assessed by questionnaire completion and objective measure of physical activity. The primary outcome - the proportion of approached patients who complete the study (incorporating rates of consent, receipt of allocated intervention and completion of both CPETs without withdrawal) – will determine progression to a full-scale RCT. Design of the proposed RCT will be informed by an embedded qualitative assessment of participant and healthcare professional involvement.

Ethics and dissemination.

This study has been approved by the East Midlands – Derby Research Ethics Committee (22/EM/0002) and the Health Research Authority. The results of this work will be disseminated academically through presentation at national and international renal meetings and via open access, peer reviewed outputs. Existing networks of renal patient groups will also be utilised to disseminate the study findings to other key stakeholders.

Trial registration number ISRCTN49033491

Sponsor: Cambridge University Hospitals NHS Foundation Trust and the University of Cambridge, Cambridge University Hospitals NHS Foundation Trust, Hills Road, Cambridge. CB2 0QQ

Protocol issue date: 21.02.2022
Version: Final 2.0

For peer review only

ARTICLE SUMMARY

Strengths and limitations of this study

- This study will undertake a gold-standard assessment of cardiorespiratory fitness following AVF ligation, with the correlate to daily activity levels provided by continuous monitoring via wearable devices.

- The inclusion of QoL evaluation will ensure the trial is patient-centred, with its outcomes immediately relevant to transplant recipients.

- The qualitative component will involve patient participants as well as assess barriers to recruitment amongst healthcare professionals.

- This feasibility study is a cost-effective and pragmatic approach to demonstrating that the proposed full-scale RCT is achievable and will likely inform further modifications of the RCT design to ensure its successful delivery.

- The relatively small numbers required for the feasibility study will limit the ability to identify a threshold value for fistula flow rate that discriminates 'high-flow' from 'low-flow' states.

For peer review only

INTRODUCTION

For most people with kidney failure, transplantation is the best form of treatment, and offers clear survival, quality of life, and cost benefits over the alternative option of continued dialysis (1-4). Reflecting this, there are now 41,000 people in the UK with a working kidney transplant, which is more than are currently receiving dialysis (5). Nevertheless, when compared to the age-matched general population, life expectancy with a working transplant remains poorer (6, 7), with the incidence of cardiovascular disease in kidney transplant patients almost five times greater than the age-matched general population (8). Consequently, recipient death with a functioning graft is now the most common cause of graft loss (9). The underlying conditions responsible for renal disease (most notably diabetes or hypertension) have well documented cardiac sequelae, and additional transplant-specific factors (such as the metabolic consequences of immunosuppression) also increase cardiovascular risk. However, arteriovenous fistulas that are created for dialysis access prior to transplantation may also have profound, and long-lasting, haemodynamic consequences, and thus may contribute to long-term cardiac mortality in kidney transplant recipients.

Arteriovenous fistulas (AVFs) are the optimal vascular access for providing haemodialysis for patients with end stage renal disease (ESRD). Compared to the alternatives; either a central venous catheter or a synthetic arteriovenous graft; AVFs last longer and are associated with improved quality of life (10, 11) and a lower incidence of sepsis-related deaths (12, 13). UK Renal Association guidelines accordingly recommend AVFs as first choice for haemodialysis access (14).

Nevertheless, AVFs have profound haemodynamic consequences. As an AVF 'matures' and the vein enlarges, the blood flow through it increases tremendously, and flows of 2-3 litres/minute are not uncommon (15). The drop in systemic vascular resistance triggers neurohormonal responses that increase cardiac contractility and circulating volume, leading to increased ventricular preload, stroke volume and cardiac output. This results in left ventricular (LV) hypertrophy and dilatation, and in severe cases may cause high-output cardiac failure (16-18). Even for relatively fit individuals, the cardiac remodelling that occurs following AVF formation likely increases their cardiovascular risk (19, 20).

There is increasing evidence that AVF disconnection improves cardiac structure in stable renal transplant recipients (21). It is however not yet clear if doing so will reduce their cardiovascular disease burden. The only randomised controlled trial (RCT) to date (22) examines the impact of AVF ligation on LV mass using cardiac MRI, and although noteworthy, has its limitations as LV mass is not necessarily reflective of functional capacity, and it has as yet not engendered a change in clinical practice. This is also reflected in the attitudes of transplant patients and their clinicians who often hold strong views about whether their AVF should be preserved or ligated. Stronger evidence is therefore required to guide patient choice and to determine whether fistula disconnection should become standard care following transplantation.

Cardiorespiratory fitness (as objectively measured by cardiopulmonary exercise testing (CPET)) provides a more accurate assessment of functional capacity than imaging studies such as cardiac MRI (23), and is a strong independent predictor of both cardiovascular risk and all-cause mortality in various patient groups, including those with ESRD (24-29). Peak oxygen uptake (VO_2, the principal CPET index) and its percentage predicted for age, sex, and weight, is the gold standard to objectively assess functional limitations, with small improvements in peak VO_2 conferring a dramatic reduction in risk of cardiovascular death (30). Importantly, quality of life (QoL) correlates with peak VO_2 for a variety of cardiac diseases (31-33), and for the individual, increases in peak VO_2 improve functional ability and QoL (34-36).

By combining rigorous assessment of cardiorespiratory fitness with activity monitoring and QoL assessment, we aim to objectively detail how AVF disconnection changes the individual's physical capacity and assess its impact on their general well-being. The feasibility study detailed in this protocol represents the first step in achieving this goal.

For peer review only - http://bmjopen.bmj.com/site/about/guidelines.xhtml

OBJECTIVES

1. To conduct a feasibility study involving six centres, that mirrors a proposed full-scale RCT, with predefined cut-offs with regards patient recruitment and retention rates to justify progression to the larger RCT.

2. To understand patients' and healthcare professionals' perceived acceptability of the proposed trial design and processes.

3. To assess feasibility and acceptability of CPET in the kidney transplant population, as judged by the proportion of participants who successfully complete both tests.

4. To assess patient compliance with wearing a wrist-worn accelerometer (physical activity measurement).

Our longer-term objective is to use the findings regarding recruitment and retention from this feasibility study to inform the design and powering of a future multicentre RCT that will test the hypothesis:

- In stable renal transplant patients, fistula disconnection improves cardiorespiratory fitness, thereby increasing patients' activity levels and improving quality of life.

METHODS AND ANALYSIS

Study design

This interventional, multicentre feasibility study will randomise forty patients in a 1:1 ratio to the intervention (fistula ligation) or to continued standard care (observance of fistula, with intervention only when clinically indicated), stratified by the fistula site (wrist or elbow) and the binary variable fistula flow rate (high or low). The overall study design is outlined in Figure 1.

Study setting

The study will be run in six NHS hospitals with a vascular access service in England and Scotland: Addenbrooke's Hospital, Cambridge; Royal Free Hospital, London; Queen Elizabeth University Hospital, Glasgow; John Radcliffe Hospital & Churchill Hospitals, Oxford; University Hospital, Coventry; Freeman Hospital, Newcastle. The study will take place from September 2021 until August 2023.

Sample selection

Participants will be considered eligible for enrolment if they fulfil the inclusion and exclusion criteria detailed below. Eligible transplant recipients will be identified by the local clinical team and approached either at their routine transplant follow-up clinic appointment or by post, with full details of the trial procedures and assessments provided at the initial screening visit. At this stage, consenting patients, as well as a small number who decline to take part in the trial, will also be invited to additionally participate in qualitative interviews exploring barriers to recruitment; this will not be a mandatory requirement for trial participation. Identifying and approaching healthcare professionals to participate in qualitative interviews will be the responsibility of the qualitative research team, drawing on their own observations from site visits and communications with the research team.

Participant eligibility

Inclusion criteria

1. Kidney transplant recipient aged 16 years or older

2. At least one year from kidney transplantation and with a patent AVF

3. Stable graft function (eGFR > 35mL/min/1.73m^2 (calculated by CKD-EPI estimation (37)) and without recent rejection episode or recent decline in graft function)

4. Adequate English to understand the study information by verbal explanation and the written participant information sheet.

For peer review only - http://bmjopen.bmj.com/site/about/guidelines.xhtml

5. Capacity to provide full informed consent

Exclusion criteria

1. Patients lacking capacity.

2. Those in whom CPET is contraindicated, as per national guidance

3. Those considered to have no further first-line options for AVF creation at their wrist or elbow, as assessed by a vascular access specialist.

4. Patients in whom AVF disconnection is indicated on clinical grounds.

Intervention

This trial will have one standard care control arm and one intervention arm, as described below.

Control: Standard care

This will involve continued conservative management, with fistula disconnection only performed if clinically indicated.

Intervention: Fistula disconnection surgery

This comprises either surgical division and oversewing of the fistula vein at the site of the original anastomosis on the artery, or disconnection of the fistula vein entirely from the artery at the anastomotic site. The operation may also include excision of the venous outflow segment if particularly enlarged or aneurysmal. The procedure may be performed under local, regional, or general anaesthesia, typically as a day-case.

Outcomes

For this feasibility study, the primary outcome measure will be the proportion of approached patients who complete the study, defined as the proportion of eligible approached patients that consent, receive the allocated intervention, and complete both CPET tests without withdrawing.

Secondary outcomes are sub-divided into: those related to the feasibility of the study; clinical measures that will be measured at baseline and six months following intervention, and the differences in these values; and safety outcome measures occurring between study consent and the six-month assessment visit.

Feasibility outcome Measures:

1. Proportion of approached patients who consent to participation
2. Proportion of participants who receive the allocated intervention
3. Proportion of participants who complete both CPETs
4. Proportion of participants who did not withdraw
5. Proportion of participants who received the allocated intervention and completed both CPETs without withdrawing
6. Proportion of participants who were compliant with wearing wrist accelerometers
7. Rate of patient recruitment at each trial centre
8. Time (days) from providing consent to first CPET and from CPET to fistula disconnection
9. Patient and healthcare professional perceived acceptability of trial design and processes (qualitative assessment carried out by interviews)

Clinical outcome measures:

1. Peak VO_2 as measured during incremental CPET on a cycle ergometer.
2. Physical functioning domain score of Short Form-36 Quality of Life questionnaire (38)
3. Activity levels (mean daily Euclidean norm minus one (ENMO)) as calculated from accelerometry data collected from provided wrist-worn accelerometer device over seven-day period at baseline and following six-month assessment
4. Serum N-terminal pro B-type natriuretic peptide (NT-proBNP) value
5. Office blood pressure

For peer review only

6. Additional CPET indices: ventilatory anaerobic threshold (VAT), endurance time, peak workload, O_2 pulse, VO_2/ work rate slope (VO_2/WR), minute ventilation/ CO_2 output slope (VE/VCO_2), heart rate/VO_2 slope (HR/VO_2), perceived exertion rating (Borg scale)
7. Additional domain scores of the Short Form-36 QoL questionnaire (38): physical role limitation, emotional role limitation, energy/ fatigue, emotional wellbeing, social functioning, pain, general health, perceived health change
8. Kidney transplant function (eGFR by CKD-EPI estimation (37))
9. Fistula-related symptoms reported in modified Vascular Access Specific QoL questionnaire (39) (for all participants at enrolment, and at six months for cohort randomised to standard care)
10. Proportion of participants with at least one major adverse cardiovascular event (MACE) (40): cardiovascular death; ischaemic cardiovascular event (myocardial infarction, percutaneous coronary intervention, coronary artery bypass graft); hospitalisation for heart failure at any time during the six-month study period

Safety outcome measures:
1. Surgical complications from fistula disconnection (number in each Clavien-Dindo classification (41))
2. Fistula-related complications in the standard care group
3. Transplant failure (return to permanent dialysis or re-transplantation)
4. Hospitalisation (number of hospitalisations and cumulative days in hospital)
5. Patient death (all-cause)

Qualitative sub-study

We will integrate qualitative research throughout the feasibility study, with the overall aim of identifying and understanding issues that might threaten the viability of a full-scale future RCT. This includes issues of recruitment and informed consent, and acceptability of the interventions and proposed outcome measure assessments.

The specific objectives of the integrated qualitative research are to:

i. Investigate healthcare professionals' perspectives on the trial design, including perceptions of equipoise, views on the eligibility criteria, relevance of the primary and secondary outcomes, and approaches to measuring these.
ii. Understand the acceptability of randomisation from patients' perspectives, with a focus on reasons for accepting/declining trial participation and factors that influenced these decisions.
iii. Explore patients' experiences of trial participation, including perceived acceptability of being in the intervention/control arms and undergoing outcome assessments (e.g. CPET, wearing accelerometers).

We will address the above objectives by conducting semi-structured interviews with healthcare professionals and patients who accept and decline trial participation. Both healthcare professionals and patients will be sampled across all six sites. We anticipate up to 40 interviews will be conducted (20 patients, 20 clinical professionals) over the two-year study period.

Progression to full-scale RCT

This feasibility study aims to test the processes of a proposed future multicentre RCT and therefore we will utilise a traffic-light approach to determine trial progression according to the following indices:

- Patient participation:
  - Green: > 40% of approached patients consent to trial participation
  - Amber: Between 30% and 40% of approached patients consent
  - Red: < 30% of patients consent

- Adherence to randomisation and study completion:
  - Green: > 85% of enrolled participants received the allocated intervention and completed both CPETs without withdrawing
  - Amber: Between 75% and 85% completion rate
  - Red: < 75% completion rate

We anticipate that a green light for the above indices will realise patient recruitment and retention rates within a future full-scale RCT to enable scheduled completion. In response to an amber trigger, the Trial

For peer review only

Management Group (TMG) will develop strategies that specifically respond to potential issues with patient participation that have been highlighted by the qualitative analysis. We would not proceed to a full-scale trial if a red signal is triggered.

During this feasibility study, we expect that, once open, each of the participating centres recruits a minimum of one patient per month and that fistula disconnection is performed within 62 days of CPET. If not met, the TMG will attempt to identify and address systematic problems that may hinder the main RCT.

Sample size
As this is a feasibility study, formal sample size calculation is not required. We will therefore aim to recruit forty patients (in keeping with recommendations (42)), twenty in each arm, with progression to a future RCT dependent on meeting set criteria relating to participant recruitment and retention rates as above. If the percentage of patients completing the study is 85%, 40 patients will give a 95% confidence interval of (70.2, 94.3).

Randomisation
Randomisation of consenting participants will be stratified according to fistula site (wrist or elbow) and fistula volume flow (low or high), as determined by a pre-randomisation Doppler ultrasound of the fistula, and allocated in a 1:1 ratio to the intervention (surgical disconnection of fistula) or to standard care (continued conservative management) using an interactive web response system provided by Sealed Envelope. Fistula flow rate will be measured during baseline assessment visits and categorised as either low flow (wrist AVF: ≤800 mL/min, elbow AVF: ≤1400 mL/min) or high flow (wrist AVF: >800 mL/min, elbow AVF: >1400 mL/min). The research team will confirm to the clinical and surgical teams which arm of the trial the participant has been randomised to, with the intervention being performed by a vascular access/transplant surgeon within 62 days of completion of baseline assessments. Given the surgical nature of the intervention, there will be no blinding in this trial with all participants, clinical and research team members aware of which trial arm is assigned at randomisation.

Statistical methods
The analysis of the primary outcome of the proportion of approached patients who complete the study successfully will include the proportion of eligible approached patients who consented, received the allocated intervention, completed both CPET tests and did not withdraw, with 95% confidence intervals. As part of the intention-to-treat approach, those who have graft failure, die, or are lost to follow-up will be included in the analysis of the primary outcome as not completing the study successfully. The component proportions and the corresponding 95% confidence intervals of the primary outcome will be presented as secondary outcomes.

The secondary feasibility outcomes of recruitment rate by trial site and the proportion of participants who were compliant with wearing wrist accelerometers, and their 95% confidence intervals, will also be presented. Compliance to wearing the monitor will be assessed by measuring wear time. The days from providing consent to first CPET and the days from CPET to fistula disconnection will be summarised, and the median and inter-quartile range presented. Patient and healthcare professional perceived acceptability of the trial design and processes will be analysed using qualitative analysis.

Descriptive statistics of the secondary clinical outcomes will be presented for each arm separately, at baseline, the six-month assessment, and the average change over time, with 95% confidence intervals. Peak VO_2 will also be presented by fistula site and according to pre-specified flow rate categories. Very limited hypothesis testing will be conducted but mixed linear regression will be used to examine any difference in peak VO_2 at six months between the two treatment arms, with adjustment for fistula site, flow rate, baseline peak VO_2 and centre. Fistula-related symptoms will be assessed using the modified VASQoL questionnaire for all participants at enrolment and at six months for the standard care arm and compared where applicable. The proportion of participants who experienced at least one MACE by six months will be presented (alongside 95% confidence intervals), and details of all reported MACEs listed. In addition, summaries of all secondary safety outcomes will be presented by arm.

For peer review only

PATIENT AND PUBLIC INVOLVEMENT

The views of both kidney transplant patients and members of the public were sought during the design of this study, both through Patient and Public Advisory Group (PPAG) meetings and distributed surveys, with final oversight from local kidney patient groups. Key aspects of the study protocol were developed following these discussions with both adaptation to the study inclusion criteria and greater emphasis placed on the incorporation of QoL assessment and daily functioning.

The feasibility study has been designed such that continued patient involvement will be a core aspect, with the integrated qualitative research aimed at capturing the participant experience and generating insight into the factors that may undermine recruitment and threaten the viability of a full-scale RCT. We have included a patient representative as part of the TMG and will also conduct six-monthly PPI meetings during the feasibility study, with lay-person involvement, to moderate discussions with patient and clinicians on their views of trial participation.

We have already established close links with local kidney patient groups at each of the trial centres; the qualitative analysis will be shared with these groups; and we anticipate that the ensuing discussions with both patients and public will promote awareness of the study, facilitate patient recruitment and retention as well as support the dissemination of results and design of the subsequent RCT.

ETHICS AND DISSEMINATION

This study has been approved by the East Midlands – Derby Research Ethics Committee (22/EM/0002) and the Health Research Authority. Research will be conducted in compliance with the approved protocol, the Declaration of Helsinki (2013), the Principles of Good Clinical Practice, the UK Data Protection Act, the UK Framework for Health and Social Care Research and any other applicable national regulations. Written informed consent will be obtained from the participants by a suitably qualified member of the clinical/research team who has received trial training. The patient's right to refuse participation in the trial and withdraw from the study at any time and without reason will be respected, without prejudice to their further treatment.

The results of this trial will be presented to academic and non-academic groups with PPAG involvement in disseminating the study findings into the public domain. Open access, peer reviewed academic outputs and research reports together with associated summaries and key findings will be produced and held on the study website.

ACCESS TO DATA

Access to the final dataset will be available on request from the Chief Investigators. No identifiable data will be shared.

DISCUSSION

There is no clear guideline on the management of AVFs following kidney transplantation. However, with the known burden of cardiovascular disease amongst renal transplant recipients, and the existing evidence that AVF disconnection improves cardiac structure, there is an increasing need to establish the role of AVF ligation in improving cardiovascular outcomes. Given the varying opinions of transplant patients and their clinicians on AVF preservation versus ligation, the COBALT feasibility study is a necessary requirement prior to pursuing a definitive robust RCT. With its embedded qualitative component, this feasibility trial aims to gauge the acceptability of the proposed trial design and optimise patient recruitment, with pre-defined stop-go criteria to justify progression to a full-scale RCT.

Acknowledgements

We thank Lewis Griffiths (MRC Epidemiology Unit) for their support for the physical activity measurement collection. We also give special thanks to Andrew Norton, our patient representative, who was instrumental in helping us develop this study proposal and securing support and funding from our local kidney patient association.

Collaborators

Subhankar Paul, Arturs Fedotov (Addenbrooke's Hospital, Cambridge); Mohammad Ayaz Hossain, John Tobi Ayorinde (Royal Free Hospital, London); Joshua Barnes (Royal Papworth Hospital, Cambridge); Emma Aitken, Carolyn Thomson, Olivia Steenson, Amy Cumming, Rowan McDougall,

For peer review only

Nicola Thomson, Aileen Campbell, Steven Brown, Leigh Bainbridge, Louise McLeod, Kaye Travers, Socrates Stoumpos (Queen Elizabeth University Hospital, Glasgow); Helen Thomas, Alice Newton (NHS Blood and Transplant Clinical Trials Unit); Lucy Wallis (University of Bristol)

Author Contributions

All authors contributed to the design of the study, refinement of the study protocol and approved the final version. SK and GJP are joint chief investigators.

Funding statement

This work was supported by National Institute for Health Research (NIHR) Research for Patient Benefit Funding grant (NIHR202255) with further support from the Addenbrooke's Kidney Patient Association and Cambridge University Hospitals NHS Foundation Trust. KLR is supported by the National Institute of Health Research (NIHR) Biomedical Research Centre in Cambridge (IS-BRC-1215-20014).

Competing interests statement

None declared.

For peer review only

REFERENCES

1. National Institute for Health and Care Excellence. Renal replacement therapy and conservative management - NICE guideline [NG107]. London: NICE; 2018.
2. RA W, VB A, EL M, AO O, RE E, LY A, et al. Comparison of mortality in all patients on dialysis, patients on dialysis awaiting transplantation, and recipients of a first cadaveric transplant. The New England journal of medicine. 1999;341(23).
3. A L, P K, N P, H K, B F, C W, et al. A study of the quality of life and cost-utility of renal transplantation. Kidney international. 1996;50(1).
4. CG R, KE T, JD R, DN C. Comparison of mortality risk for dialysis patients and cadaveric first renal transplant recipients in Ontario, Canada. Journal of the American Society of Nephrology : JASN. 2000;11(5).
5. UK Renal Registry. UK Renal Registry 21st Annual Report – data to 31/12/2017. Bristol, UK; 2019.
6. Karim A, Farrugia D, Cheshire J, Mahboob S, Begaj I, Ray D, et al. Recipient age and risk for mortality after kidney transplantation in England. Transplantation. 2014;97(8):832-8.
7. Kramer A, Boenink R, Noordzij M, Bosdriesz JR, Stel VS, Beltrán P, et al. The ERA-EDTA Registry Annual Report 2017: a summary. Clinical Kidney Journal. 2020.
8. Jardine AG, Gaston RS, Fellstrom BC, Holdaas H. Prevention of cardiovascular disease in adult recipients of kidney transplants. Lancet. 2011;378(9800):1419-27.
9. Arce CM, Lenihan CR, Montez-Rath ME, Winkelmayer WC. Comparison of longer-term outcomes after kidney transplantation between Hispanic and non-Hispanic whites in the United States. Am J Transplant. 2015;15(2):499-507.
10. Domenick Sridharan N, Fish L, Yu L, Weisbord S, Jhamb M, Makaroun MS, et al. The associations of hemodialysis access type and access satisfaction with health-related quality of life. J Vasc Surg. 2018;67(1):229-35.
11. Korevaar JC, Merkus MP, Jansen MA, Dekker FW, Boeschoten EW, Krediet RT, et al. Validation of the KDQOL-SF: a dialysis-targeted health measure. Qual Life Res. 2002;11(5):437-47.
12. Pastan S, Soucie JM, McClellan WM. Vascular access and increased risk of death among hemodialysis patients. Kidney Int. 2002;62(2):620-6.
13. Polkinghorne KR, McDonald SP, Atkins RC, Kerr PG. Vascular access and all-cause mortality: a propensity score analysis. J Am Soc Nephrol. 2004;15(2):477-86.
14. Kumwenda M, Mitra S, Reid C. Vascular access for haemodialysis. UK Renal Association; 2015.
15. Aitken E, Kerr D, Geddes C, Berry C, Kingsmore D. Cardiovascular changes occurring with occlusion of a mature arteriovenous fistula. J Vasc Access. 2015;16(6):459-66.
16. Hartley JL, Sharma A, Taha L, Hestletine T. High-output cardiac failure secondary to high-output arteriovenous fistula: investigations, management and definitive treatment. BMJ Case Rep. 2020;13(2).
17. Wasse H, Singapuri MS. High-output heart failure: how to define it, when to treat it, and how to treat it. Semin Nephrol. 2012;32(6):551-7.
18. MacRae JM, Pandeya S, Humen DP, Krivitski N, Lindsay RM. Arteriovenous fistula-associated high-output cardiac failure: a review of mechanisms. Am J Kidney Dis. 2004;43(5):e17-22.
19. Levy D, Garrison RJ, Savage DD, Kannel WB, Castelli WP. Prognostic implications of echocardiographically determined left ventricular mass in the Framingham Heart Study. N Engl J Med. 1990;322(22):1561-6.
20. Tsao CW, Gona PN, Salton CJ, Chuang ML, Levy D, Manning WJ, et al. Left Ventricular Structure and Risk of Cardiovascular Events: A Framingham Heart Study Cardiac Magnetic Resonance Study. J Am Heart Assoc. 2015;4(9):e002188.
21. Zheng H, Bu S, Song Y, Wang M, Wu J, Chen J. To Ligate or Not to Ligate: A Meta-analysis of Cardiac Effects and Allograft Function following Arteriovenous Fistula Closure in Renal Transplant Recipients. Ann Vasc Surg. 2020;63:287-92.
22. Rao NN, Stokes MB, Rajwani A, Ullah S, Williams K, King D, et al. Effects of Arteriovenous Fistula Ligation on Cardiac Structure and Function in Kidney Transplant Recipients. Circulation. 2019;139(25):2809-18.
23. Society AT, Physicians ACoC. ATS/ACCP Statement on cardiopulmonary exercise testing. Am J Respir Crit Care Med. 2003;167(2):211-77.
24. Myers J, Prakash M, Froelicher V, Do D, Partington S, Atwood JE. Exercise capacity and mortality among men referred for exercise testing. N Engl J Med. 2002;346(11):793-801.

For peer review only

25. Kodama S, Saito K, Tanaka S, Maki M, Yachi Y, Asumi M, et al. Cardiorespiratory fitness as a quantitative predictor of all-cause mortality and cardiovascular events in healthy men and women: a meta-analysis. JAMA. 2009;301(19):2024-35.
26. Swank AM, Horton J, Fleg JL, Fonarow GC, Keteyian S, Goldberg L, et al. Modest increase in peak VO2 is related to better clinical outcomes in chronic heart failure patients: results from heart failure and a controlled trial to investigate outcomes of exercise training. Circ Heart Fail. 2012;5(5):579-85.
27. Ting SM, Iqbal H, Kanji H, Hamborg T, Aldridge N, Krishnan N, et al. Functional cardiovascular reserve predicts survival pre-kidney and post-kidney transplantation. J Am Soc Nephrol. 2014;25(1):187-95.
28. Sietsema KE, Amato A, Adler SG, Brass EP. Exercise capacity as a predictor of survival among ambulatory patients with end-stage renal disease. Kidney Int. 2004;65(2):719-24.
29. Ross R, Blair SN, Arena R, Church TS, Després JP, Franklin BA, et al. Importance of Assessing Cardiorespiratory Fitness in Clinical Practice: A Case for Fitness as a Clinical Vital Sign: A Scientific Statement From the American Heart Association. Circulation. 2016;134(24):e653-e99.
30. Imboden MT, Harber MP, Whaley MH, Finch WH, Bishop DL, Fleenor BS, et al. The Association between the Change in Directly Measured Cardiorespiratory Fitness across Time and Mortality Risk. Prog Cardiovasc Dis. 2019;62(2):157-62.
31. Hager A, Hess J. Comparison of health related quality of life with cardiopulmonary exercise testing in adolescents and adults with congenital heart disease. Heart. 2005;91(4):517-20.
32. Athanasopoulos LV, Dritsas A, Doll HA, Cokkinos DV. Explanation of the variance in quality of life and activity capacity of patients with heart failure by laboratory data. Eur J Cardiovasc Prev Rehabil. 2010;17(4):375-9.
33. Huff CM, Turer AT, Wang A. Correlations between physician-perceived functional status, patient-perceived health status, and cardiopulmonary exercise results in hypertrophic cardiomyopathy. Qual Life Res. 2013;22(3):647-52.
34. Eriksen L, Curtis T, Grønbæk M, Helge JW, Tolstrup JS. The association between physical activity, cardiorespiratory fitness and self-rated health. Prev Med. 2013;57(6):900-2.
35. Edelmann F, Gelbrich G, Düngen HD, Fröhling S, Wachter R, Stahrenberg R, et al. Exercise training improves exercise capacity and diastolic function in patients with heart failure with preserved ejection fraction: results of the Ex-DHF (Exercise training in Diastolic Heart Failure) pilot study. J Am Coll Cardiol. 2011;58(17):1780-91.
36. Kitzman DW, Brubaker PH, Herrington DM, Morgan TM, Stewart KP, Hundley WG, et al. Effect of endurance exercise training on endothelial function and arterial stiffness in older patients with heart failure and preserved ejection fraction: a randomized, controlled, single-blind trial. J Am Coll Cardiol. 2013;62(7):584-92.
37. Delanaye P, Mariat C. The applicability of eGFR equations to different populations. Nat Rev Nephrol. 2013;9(9):513-22.
38. Shield CF, McGrath MM, Goss TF. Assessment of health-related quality of life in kidney transplant patients receiving tacrolimus (FK506)-based versus cyclosporine-based immunosuppression. FK506 Kidney Transplant Study Group. Transplantation. 1997;64(12):1738-43.
39. Richarz S, Greenwood S, Kingsmore DB, Thomson PC, Dunlop M, Bouamrane MM, et al. Validation of a vascular access specific quality of life measure (VASQoL). J Vasc Access. 2021:11297298211046746.
40. Holdaas H, Fellström B, Jardine AG, Holme I, Nyberg G, Fauchald P, et al. Effect of fluvastatin on cardiac outcomes in renal transplant recipients: a multicentre, randomised, placebo-controlled trial. Lancet. 2003;361(9374):2024-31.
41. Dindo D, Demartines N, Clavien PA. Classification of surgical complications: a new proposal with evaluation in a cohort of 6336 patients and results of a survey. Ann Surg. 2004;240(2):205-13.
42. Lancaster GA, Dodd S, Williamson PR. Design and analysis of pilot studies: recommendations for good practice. J Eval Clin Pract. 2004;10(2):307-12.

For peer review only

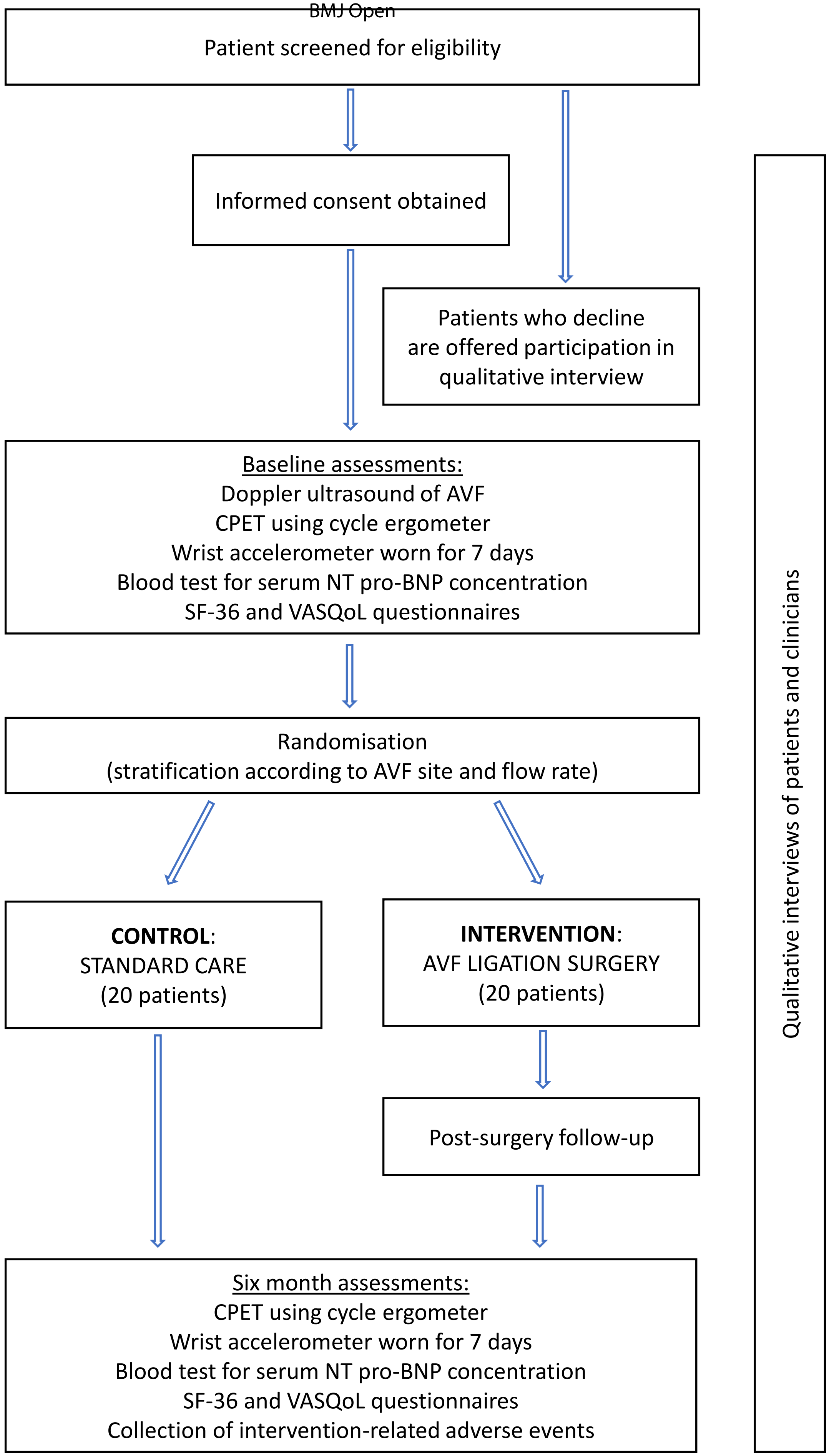

Figure 1: Overall design of COBALT study. AVF, Arteriovenous fistula CPET, Cardiopulmonary exercise test; NT pro-BNP, N-terminal pro-B-type natriuretic peptide; SF-36, Short Form 36; VASQoL, Vascular Access Specific Quality of Life

Reporting checklist for protocol of a clinical trial.

Based on the SPIRIT guidelines.

Instructions to authors

Complete this checklist by entering the page numbers from your manuscript where readers will find each of the items listed below.

Your article may not currently address all the items on the checklist. Please modify your text to include the missing information. If you are certain that an item does not apply, please write "n/a" and provide a short explanation.

Upload your completed checklist as an extra file when you submit to a journal.

In your methods section, say that you used the SPIRITreporting guidelines, and cite them as:

Chan A-W, Tetzlaff JM, Gøtzsche PC, Altman DG, Mann H, Berlin J, Dickersin K, Hróbjartsson A, Schulz KF, Parulekar WR, Krleža-Jerić K, Laupacis A, Moher D. SPIRIT 2013 Explanation and Elaboration: Guidance for protocols of clinical trials. BMJ. 2013;346:e7586

		Reporting Item	Page Number
**Administrative information**			
Title	#1	Descriptive title identifying the study design, population, interventions, and, if applicable, trial acronym	2

For peer review only

Trial registration	#2a	Trial identifier and registry name. If not yet registered, name of intended registry	4
Trial registration: data set	#2b	All items from the World Health Organization Trial Registration Data Set	4
Protocol version	#3	Date and version identifier	4
Funding	#4	Sources and types of financial, material, and other support	12
Roles and responsibilities: contributorship	#5a	Names, affiliations, and roles of protocol contributors	2
Roles and responsibilities: sponsor contact information	#5b	Name and contact information for the trial sponsor	4
Roles and responsibilities: sponsor and funder	#5c	Role of study sponsor and funders, if any, in study design; collection, management, analysis, and interpretation of data; writing of the report; and the decision to submit the report for publication, including whether they will have ultimate authority over any of these activities	n/a
Roles and responsibilities: committees	#5d	Composition, roles, and responsibilities of the coordinating centre, steering committee, endpoint adjudication committee, data management team, and	n/a

For peer review only

		other individuals or groups overseeing the trial, if applicable (see Item 21a for data monitoring committee)	
**Introduction**			
Background and rationale	#6a	Description of research question and justification for undertaking the trial, including summary of relevant studies (published and unpublished) examining benefits and harms for each intervention	6
Background and rationale: choice of comparators	#6b	Explanation for choice of comparators	6
Objectives	#7	Specific objectives or hypotheses	7
Trial design	#8	Description of trial design including type of trial (eg, parallel group, crossover, factorial, single group), allocation ratio, and framework (eg, superiority, equivalence, non-inferiority, exploratory)	7
**Methods: Participants, interventions, and outcomes**			
Study setting	#9	Description of study settings (eg, community clinic, academic hospital) and list of countries where data will be collected. Reference to where list of study sites can be obtained	7

For peer review only

Eligibility criteria	#10	Inclusion and exclusion criteria for participants. If applicable, eligibility criteria for study centres and individuals who will perform the interventions (eg, surgeons, psychotherapists)	7
Interventions: description	#11a	Interventions for each group with sufficient detail to allow replication, including how and when they will be administered	8
Interventions: modifications	#11b	Criteria for discontinuing or modifying allocated interventions for a given trial participant (eg, drug dose change in response to harms, participant request, or improving / worsening disease)	n/a
Interventions: adherance	#11c	Strategies to improve adherence to intervention protocols, and any procedures for monitoring adherence (eg, drug tablet return; laboratory tests)	n/a
Interventions: concomitant care	#11d	Relevant concomitant care and interventions that are permitted or prohibited during the trial	n/a
Outcomes	#12	Primary, secondary, and other outcomes, including the specific measurement variable (eg, systolic blood pressure), analysis metric (eg, change from baseline, final value, time to event), method of aggregation (eg, median, proportion), and time point for each outcome. Explanation of the clinical relevance of chosen efficacy and harm outcomes is strongly recommended	8

For peer review only

Participant timeline	#13	Time schedule of enrolment, interventions (including any run-ins and washouts), assessments, and visits for participants. A schematic diagram is highly recommended (see Figure)	See Figure
Sample size	#14	Estimated number of participants needed to achieve study objectives and how it was determined, including clinical and statistical assumptions supporting any sample size calculations	9
Recruitment	#15	Strategies for achieving adequate participant enrolment to reach target sample size	7
**Methods: Assignment of interventions (for controlled trials)**			
Allocation: sequence generation	#16a	Method of generating the allocation sequence (eg, computer-generated random numbers), and list of any factors for stratification. To reduce predictability of a random sequence, details of any planned restriction (eg, blocking) should be provided in a separate document that is unavailable to those who enrol participants or assign interventions	10
Allocation concealment mechanism	#16b	Mechanism of implementing the allocation sequence (eg, central telephone; sequentially numbered, opaque,	10

For peer review only

		sealed envelopes), describing any steps to conceal the sequence until interventions are assigned	
Allocation: implementation	#16c	Who will generate the allocation sequence, who will enrol participants, and who will assign participants to interventions	10
Blinding (masking)	#17a	Who will be blinded after assignment to interventions (eg, trial participants, care providers, outcome assessors, data analysts), and how	n/a
Blinding (masking): emergency unblinding	#17b	If blinded, circumstances under which unblinding is permissible, and procedure for revealing a participant's allocated intervention during the trial	n/a
**Methods: Data collection, management, and analysis**			
Data collection plan	#18a	Plans for assessment and collection of outcome, baseline, and other trial data, including any related processes to promote data quality (eg, duplicate measurements, training of assessors) and a description of study instruments (eg, questionnaires, laboratory tests) along with their reliability and validity, if known. Reference to where data collection forms can be found, if not in the protocol	8

For peer review only

Data collection plan: retention	#18b	Plans to promote participant retention and complete follow-up, including list of any outcome data to be collected for participants who discontinue or deviate from intervention protocols	n/a
Data management	#19	Plans for data entry, coding, security, and storage, including any related processes to promote data quality (eg, double data entry; range checks for data values). Reference to where details of data management procedures can be found, if not in the protocol	n/a
Statistics: outcomes	#20a	Statistical methods for analysing primary and secondary outcomes. Reference to where other details of the statistical analysis plan can be found, if not in the protocol	8
Statistics: additional analyses	#20b	Methods for any additional analyses (eg, subgroup and adjusted analyses)	n/a
Statistics: analysis population and missing data	#20c	Definition of analysis population relating to protocol non-adherence (eg, as randomised analysis), and any statistical methods to handle missing data (eg, multiple imputation)	n/a

Methods: Monitoring

Data monitoring: formal committee	#21a	Composition of data monitoring committee (DMC); summary of its role and reporting structure; statement of whether it is independent from the sponsor and competing interests; and reference to where further	n/a

For peer review only

		details about its charter can be found, if not in the protocol. Alternatively, an explanation of why a DMC is not needed	
Data monitoring: interim analysis	#21b	Description of any interim analyses and stopping guidelines, including who will have access to these interim results and make the final decision to terminate the trial	n/a
Harms	#22	Plans for collecting, assessing, reporting, and managing solicited and spontaneously reported adverse events and other unintended effects of trial interventions or trial conduct	n/a
Auditing	#23	Frequency and procedures for auditing trial conduct, if any, and whether the process will be independent from investigators and the sponsor	n/a
**Ethics and dissemination**			
Research ethics approval	#24	Plans for seeking research ethics committee / institutional review board (REC / IRB) approval	11
Protocol amendments	#25	Plans for communicating important protocol modifications (eg, changes to eligibility criteria, outcomes, analyses) to relevant parties (eg, investigators, REC / IRBs, trial participants, trial registries, journals, regulators)	n/a

For peer review only

Consent or assent	#26a	Who will obtain informed consent or assent from potential trial participants or authorised surrogates, and how (see Item 32)	7
Consent or assent: ancillary studies	#26b	Additional consent provisions for collection and use of participant data and biological specimens in ancillary studies, if applicable	n/a
Confidentiality	#27	How personal information about potential and enrolled participants will be collected, shared, and maintained in order to protect confidentiality before, during, and after the trial	n/a
Declaration of interests	#28	Financial and other competing interests for principal investigators for the overall trial and each study site	12
Data access	#29	Statement of who will have access to the final trial dataset, and disclosure of contractual agreements that limit such access for investigators	11
Ancillary and post trial care	#30	Provisions, if any, for ancillary and post-trial care, and for compensation to those who suffer harm from trial participation	n/a
Dissemination policy: trial results	#31a	Plans for investigators and sponsor to communicate trial results to participants, healthcare professionals, the public, and other relevant groups (eg, via publication, reporting in results databases, or other data sharing arrangements), including any publication restrictions	11

For peer review only

Dissemination policy: authorship	#31b	Authorship eligibility guidelines and any intended use of professional writers	12
Dissemination policy: reproducible research	#31c	Plans, if any, for granting public access to the full protocol, participant-level dataset, and statistical code	11

Appendices

Informed consent materials	#32	Model consent form and other related documentation given to participants and authorised surrogates	n/a
Biological specimens	#33	Plans for collection, laboratory evaluation, and storage of biological specimens for genetic or molecular analysis in the current trial and for future use in ancillary studies, if applicable	n/a

The SPIRIT Explanation and Elaboration paper is distributed under the terms of the Creative Commons Attribution License CC-BY-NC. This checklist was completed on 20. August 2022 using https://www.goodreports.org/, a tool made by the EQUATOR Network in collaboration with Penelope.ai

For peer review only

BMJ Open

Cardiorespiratory Optimisation By Arteriovenous fistula Ligation after renal Transplantation (COBALT): study protocol for a multicentre randomised interventional feasibility trial

Journal:	*BMJ Open*
Manuscript ID	bmjopen-2022-067668.R1
Article Type:	Protocol
Date Submitted by the Author:	11-Oct-2022
Complete List of Authors:	Surendrakumar, Veena; University Hospitals Birmingham NHS Foundation Trust<br>Aitken, Emma; Queen Elizabeth University Hospital; University of Glasgow, Institute of Cardiovascular and Medical Sciences<br>Mark, Patrick; University of Glasgow, Institute of Cardiovascular and Medical Sciences<br>Motallebzadeh, Reza; Royal Free London NHS Foundation Trust; University College London<br>Hunter, James; University Hospitals Coventry and Warwickshire NHS Trust; University of Oxford<br>Amer, Aimen; Newcastle Upon Tyne Hospitals NHS Foundation Trust<br>Summers, Dominic ; Cambridge University Hospitals NHS Foundation Trust<br>Rennie, Kirsten ; University of Cambridge, MRC Epidemiology Unit<br>Rooshenas, Leila; University of Bristol<br>Garbi, Madalina ; Royal Papworth Hospital NHS Foundation Trust<br>Sylvester, Karl; Cambridge University Hospitals NHS Foundation Trust<br>Hudson, Cara; NHS Blood and Transplant Organ Donation and Transplantation Directorate, Statistics and Clinical Studies<br>Banks, Jennifer; NHS Blood and Transplant Organ Donation and Transplantation Directorate, Statistics and Clinical Studies<br>Sidders, Anna; NHSBT Clinical Trials Unit, Clinical Trials Unit<br>Norton, Andrew; Addenbrooke's Kidney Patients Association<br>Slater, Matthew; Cambridge University Hospitals NHS Foundation Trust<br>Bartlett, Matthew; Royal Free London NHS Foundation Trust, Vascular Studies<br>Knight, Simon; University of Oxford<br>Pettigrew, Gavin ; University of Cambridge, Surgery; Cambridge University Hospitals NHS Foundation Trust<br>trial group, COBALT; NHS Blood and Transplant Organ Donation and Transplantation Directorate
**Primary Subject Heading**:	Renal medicine
Secondary Subject Heading:	Surgery, Cardiovascular medicine
Keywords:	Renal transplantation < NEPHROLOGY, Transplant surgery < SURGERY,

	Transplant medicine < INTERNAL MEDICINE

SCHOLARONE™
Manuscripts

Cardiorespiratory Optimisation By Arteriovenous fistula Ligation after renal Transplantation (COBALT): study protocol for a multicentre randomised interventional feasibility trial

Authors
Veena Surendrakumar
University Hospitals Birmingham NHS Foundation Trust
veena.surendrakumar@nhs.net

Emma Aitken
Queen Elizabeth University Hospital, Glasgow; Institute of Cardiovascular and Medical Sciences, University of Glasgow
emma.aitken@ggc.scot.nhs.uk

Patrick B Mark
Institute of Cardiovascular and Medical Sciences, University of Glasgow
Patrick.Mark@glasgow.ac.uk

Reza Motallebzadeh
Royal Free London NHS Foundation Trust and University College London
reza.motallebzadeh@nhs.net

James P Hunter
University of Oxford and University Hospitals Coventry and Warwickshire NHS Trust
james.hunter@nds.ox.ac.uk

Aimen Amer
Newcastle Upon Tyne Hospitals NHS Foundation Trust
aimen.amer@nhs.net

Dominic Summers
Cambridge University Hospitals NHS Foundation Trust
dms39@cam.ac.uk

Kirsten L Rennie
MRC Epidemiology Unit, University of Cambridge
Kirsten.Rennie@mrc-epid.cam.ac.uk

Leila Rooshenas
University of Bristol
leila.Rooshenas@bristol.ac.uk

Madalina Garbi
Royal Papworth Hospital NHS Foundation Trust
madalina.garbi@nhs.net

Karl Sylvester
Cambridge University Hospitals NHS Foundation Trust
karl.sylvester@nhs.net

Cara Hudson
NHS Blood and Transplant Clinical Trials Unit
Cara.Hudson@nhsbt.nhs.uk

Jenni Banks
NHS Blood and Transplant Clinical Trials Unit
jenni.banks@nhsbt.nhs.uk

For peer review only

Anna Sidders
NHS Blood and Transplant Clinical Trials Unit
Anna.Sidders@nhsbt.nhs.uk

Andrew Norton
Addenbrooke's Kidney Patients Association
andrew.j.norton@pdmassociates.co.uk

Matthew Slater
Cambridge University Hospitals NHS Foundation Trust
matthewslater@nhs.net

Matthew Bartlett
Royal Free London NHS Foundation Trust
matthew.bartlett@nhs.net

Simon Knight
University of Oxford
simon.knight@nds.ox.ac.uk

Gavin J Pettigrew
University of Cambridge
gjp25@cam.ac.uk

COBALT trial group
COBALT@nhsbt.nhs.uk

KEYWORDS
Arteriovenous fistula
Ligation
Kidney transplant
Cardiopulmonary exercise testing
Quality of life

WORD COUNT
3272 words

Corresponding Author:
Mr Gavin J Pettigrew
Department of Surgery
University of Cambridge
Box 202, Level E9 Addenbrooke's Hospital
Hills Road
Cambridge
CB2 0QQ

gjp25@cam.ac.uk

Tel: 01223 336976

For peer review only

ABSTRACT

Introduction

Cardiovascular events are a major cause of mortality following successful kidney transplantation. Arteriovenous fistulas (AVFs) are considered the best option for haemodialysis, but may contribute to this excess mortality because they promote adverse cardiac remodelling and ventricular hypertrophy. This raises the question whether recipients with a well-functioning kidney transplant should undergo elective AVF ligation.

Methods and analysis

The COBALT feasibility study is a multicentre interventional randomised controlled trial (RCT) that will randomise renal transplant patients with stable graft function and a working AVF on a 1:1 basis to standard care (continued conservative management) or to AVF ligation. All patients will perform cardiopulmonary exercise testing (CPET) on recruitment and six months later. Daily functioning and quality of life (QoL) will be additionally assessed by questionnaire completion and objective measure of physical activity. The primary outcome - the proportion of approached patients who complete the study (incorporating rates of consent, receipt of allocated intervention and completion of both CPETs without withdrawal) – will determine progression to a full-scale RCT. Design of the proposed RCT will be informed by an embedded qualitative assessment of participant and healthcare professional involvement.

Ethics and dissemination.

This study has been approved by the East Midlands – Derby Research Ethics Committee (22/EM/0002) and the Health Research Authority. The results of this work will be disseminated academically through presentation at national and international renal meetings and via open access, peer reviewed outputs. Existing networks of renal patient groups will also be utilised to disseminate the study findings to other key stakeholders.

Trial registration number ISRCTN49033491

Sponsor: Cambridge University Hospitals NHS Foundation Trust and the University of Cambridge, Cambridge University Hospitals NHS Foundation Trust, Hills Road, Cambridge. CB2 0QQ

Protocol issue date: 21.02.2022
Version: Final 2.0

For peer review only

ARTICLE SUMMARY

Strengths and limitations of this study

- This study will undertake a gold-standard assessment of cardiorespiratory fitness following AVF ligation, with the correlate to daily activity levels provided by continuous monitoring via wearable devices.
- The inclusion of QoL evaluation will ensure the trial is patient-centred, with its outcomes immediately relevant to transplant recipients.
- The qualitative component will involve patient participants as well as assess barriers to recruitment amongst healthcare professionals.
- This feasibility study is a cost-effective and pragmatic approach to demonstrating that the proposed full-scale RCT is achievable and will likely inform further modifications of the RCT design to ensure its successful delivery.
- The relatively small numbers required for the feasibility study will limit the ability to identify a threshold value for fistula flow rate that discriminates 'high-flow' from 'low-flow' states.

For peer review only

INTRODUCTION

For most people with kidney failure, transplantation is the best form of treatment, and offers clear survival, quality of life, and cost benefits over the alternative option of continued dialysis (1-4). Reflecting this, there are now 41,000 people in the UK with a working kidney transplant, which is more than are currently receiving dialysis (5). Nevertheless, when compared to the age-matched general population, life expectancy with a working transplant remains poorer (6, 7), with the incidence of cardiovascular disease in kidney transplant patients almost five times greater than the age-matched general population (8). Consequently, recipient death with a functioning graft is now the most common cause of graft loss (9). The underlying conditions responsible for renal disease (most notably diabetes or hypertension) have well documented cardiac sequelae, and additional transplant-specific factors (such as the metabolic consequences of immunosuppression) also increase cardiovascular risk. However, arteriovenous fistulas that are created for dialysis access prior to transplantation may also have profound, and long-lasting, haemodynamic consequences, and thus may contribute to long-term cardiac mortality in kidney transplant recipients.

Arteriovenous fistulas (AVFs) are the optimal vascular access for providing haemodialysis for patients with end stage renal disease (ESRD). Compared to the alternatives; either a central venous catheter or a synthetic arteriovenous graft; AVFs last longer and are associated with improved quality of life (10, 11) and a lower incidence of sepsis-related deaths (12, 13). UK Renal Association guidelines accordingly recommend AVFs as first choice for haemodialysis access (14).

Nevertheless, AVFs have profound haemodynamic consequences. As an AVF 'matures' and the vein enlarges, the blood flow through it increases tremendously, and flows of 2-3 litres/minute are not uncommon (15). The drop in systemic vascular resistance triggers neurohormonal responses that increase cardiac contractility and circulating volume, leading to increased ventricular preload, stroke volume and cardiac output. This results in left ventricular (LV) hypertrophy and dilatation, and in severe cases may cause high-output cardiac failure (16-18). Even for relatively fit individuals, the cardiac remodelling that occurs following AVF formation likely increases their cardiovascular risk (19, 20).

There is increasing evidence that AVF disconnection improves cardiac structure in stable renal transplant recipients (21). It is however not yet clear if doing so will reduce their cardiovascular disease burden. The only randomised controlled trial (RCT) to date (22) examines the impact of AVF ligation on LV mass using cardiac MRI, and although noteworthy, has its limitations as LV mass is not necessarily reflective of functional capacity, and it has as yet not engendered a change in clinical practice. This is also reflected in the attitudes of transplant patients and their clinicians who often hold strong views about whether their AVF should be preserved or ligated. Stronger evidence is therefore required to guide patient choice and to determine whether fistula disconnection should become standard care following transplantation.

Cardiorespiratory fitness (as objectively measured by cardiopulmonary exercise testing (CPET)) provides a more accurate assessment of functional capacity than imaging studies such as cardiac MRI (23), and is a strong independent predictor of both cardiovascular risk and all-cause mortality in various patient groups, including those with ESRD (24-29). Peak oxygen uptake (VO_2, the principal CPET index) and its percentage predicted for age, sex, and weight, is the gold standard to objectively assess functional limitations, with small improvements in peak VO_2 conferring a dramatic reduction in risk of cardiovascular death (30). Importantly, quality of life (QoL) correlates with peak VO_2 for a variety of cardiac diseases (31-33), and for the individual, increases in peak VO_2 improve functional ability and QoL (34-36).

By combining rigorous assessment of cardiorespiratory fitness with activity monitoring and QoL assessment, we aim to objectively detail how AVF disconnection changes the individual's physical capacity and assess its impact on their general well-being. The feasibility study detailed in this protocol represents the first step in achieving this goal.

For peer review only - http://bmjopen.bmj.com/site/about/guidelines.xhtml

OBJECTIVES

1. To conduct a feasibility study involving six centres, that mirrors a proposed full-scale RCT, with predefined cut-offs with regards patient recruitment and retention rates to justify progression to the larger RCT.

2. To understand patients' and healthcare professionals' perceived acceptability of the proposed trial design and processes.

3. To assess feasibility and acceptability of CPET in the kidney transplant population, as judged by the proportion of participants who successfully complete both tests.

4. To assess patient compliance with wearing a wrist-worn accelerometer (physical activity measurement).

Our longer-term objective is to use the findings regarding recruitment and retention from this feasibility study to inform the design and powering of a future multicentre RCT that will test the hypothesis:

- In stable renal transplant patients, fistula disconnection improves cardiorespiratory fitness, thereby increasing patients' activity levels and improving quality of life.

METHODS AND ANALYSIS

Study design

This interventional, multicentre feasibility study will randomise forty patients in a 1:1 ratio to the intervention (fistula ligation) or to continued standard care (observance of fistula, with intervention only when clinically indicated), stratified by the fistula site (wrist or elbow) and the binary variable fistula flow rate (high or low). The overall study design is outlined in Figure 1.

Study setting

The study will take place in six NHS hospitals with a vascular access service in England and Scotland: Addenbrooke's Hospital, Cambridge; Royal Free Hospital, London; Queen Elizabeth University Hospital, Glasgow; John Radcliffe Hospital & Churchill Hospitals, Oxford; University Hospital, Coventry; Freeman Hospital, Newcastle. The study will run from 1st September 2021 until 31st July 2023, with recruitment taking place between 1st March 2022 until 30th November 2022.

Sample selection

Participants will be considered eligible for enrolment if they fulfil the inclusion and exclusion criteria detailed below. Eligible transplant recipients will be identified by the local clinical team and approached either at their routine transplant follow-up clinic appointment or by post, with full details of the trial procedures and assessments provided at the initial screening visit. At this stage, consenting patients, as well as a small number who decline to take part in the trial, will also be invited to additionally participate in qualitative interviews exploring barriers to recruitment; this will not be a mandatory requirement for trial participation. Identifying and approaching healthcare professionals to participate in qualitative interviews will be the responsibility of the qualitative research team, drawing on their own observations from site visits and communications with the research team. Informed written consent will be obtained from all trial participants (supplementary files).

Participant eligibility

Inclusion criteria

1. Kidney transplant recipient aged 16 years or older

2. At least one year from kidney transplantation and with a patent AVF

3. Stable graft function (eGFR > 35mL/min/1.73m^2 (calculated by CKD-EPI estimation (37)) and without recent rejection episode or recent decline in graft function)

For peer review only

4. Adequate English to understand the study information by verbal explanation and the written participant information sheet.

5. Capacity to provide full informed consent

Exclusion criteria

1. Patients lacking capacity.

2. Those in whom CPET is contraindicated, as per national guidance

3. Those considered to have no further first-line options for AVF creation at their wrist or elbow, as assessed by a vascular access specialist.

4. Patients in whom AVF disconnection is indicated on clinical grounds.

Intervention

This trial will have one standard care control arm and one intervention arm, as described below.

Control: Standard care

This will involve continued conservative management, with fistula disconnection only performed if clinically indicated.

Intervention: Fistula disconnection surgery

This comprises either surgical division and oversewing of the fistula vein at the site of the original anastomosis on the artery, or disconnection of the fistula vein entirely from the artery at the anastomotic site. The operation may also include excision of the venous outflow segment if particularly enlarged or aneurysmal. The procedure may be performed under local, regional, or general anaesthesia, typically as a day-case.

Outcomes

For this feasibility study, the primary outcome measure will be the proportion of approached patients who complete the study, defined as the proportion of eligible approached patients that consent, receive the allocated intervention, and complete both CPET tests without withdrawing.

Secondary outcomes are sub-divided into: those related to the feasibility of the study; clinical measures that will be measured at baseline and six months following intervention, and the differences in these values; and safety outcome measures occurring between study consent and the six-month assessment visit.

Feasibility outcome Measures:

1. Proportion of approached patients who consent to participation
2. Proportion of participants who receive the allocated intervention
3. Proportion of participants who complete both CPETs
4. Proportion of participants who did not withdraw
5. Proportion of participants who received the allocated intervention and completed both CPETs without withdrawing
6. Proportion of participants who were compliant with wearing wrist accelerometers
7. Rate of patient recruitment at each trial centre
8. Time (days) from providing consent to first CPET and from CPET to fistula disconnection
9. Patient and healthcare professional perceived acceptability of trial design and processes (qualitative assessment carried out by interviews)

Clinical outcome measures:

1. Peak VO_2 as measured during incremental CPET on a cycle ergometer.
2. Physical functioning domain score of Short Form-36 Quality of Life questionnaire (38)
3. Activity levels (mean daily Euclidean norm minus one (ENMO)) as calculated from accelerometry data collected from provided wrist-worn accelerometer device over seven-day period at baseline and following six-month assessment
4. Serum N-terminal pro B-type natriuretic peptide (NT-proBNP) value

For peer review only

5. Office blood pressure
6. Additional CPET indices: ventilatory anaerobic threshold (VAT), endurance time, peak workload, O_2 pulse, VO_2/ work rate slope (VO_2/WR), minute ventilation/ CO_2 output slope (VE/VCO_2), heart rate/VO_2 slope (HR/VO_2), perceived exertion rating (Borg scale)
7. Additional domain scores of the Short Form-36 QoL questionnaire (38): physical role limitation, emotional role limitation, energy/ fatigue, emotional wellbeing, social functioning, pain, general health, perceived health change
8. Kidney transplant function (eGFR by CKD-EPI estimation (37))
9. Fistula-related symptoms reported in modified Vascular Access Specific QoL questionnaire (39) (for all participants at enrolment, and at six months for cohort randomised to standard care)
10. Proportion of participants with at least one major adverse cardiovascular event (MACE) (40): cardiovascular death; ischaemic cardiovascular event (myocardial infarction, percutaneous coronary intervention, coronary artery bypass graft); hospitalisation for heart failure at any time during the six-month study period

Safety outcome measures:
1. Surgical complications from fistula disconnection (number in each Clavien-Dindo classification (41))
2. Fistula-related complications in the standard care group
3. Transplant failure (return to permanent dialysis or re-transplantation)
4. Hospitalisation (number of hospitalisations and cumulative days in hospital)
5. Patient death (all-cause)

Qualitative sub-study
We will integrate qualitative research throughout the feasibility study, with the overall aim of identifying and understanding issues that might threaten the viability of a full-scale future RCT. This includes issues of recruitment and informed consent, and acceptability of the interventions and proposed outcome measure assessments.

The specific objectives of the integrated qualitative research are to:
i. Investigate healthcare professionals' perspectives on the trial design, including perceptions of equipoise, views on the eligibility criteria, relevance of the primary and secondary outcomes, and approaches to measuring these.
ii. Understand the acceptability of randomisation from patients' perspectives, with a focus on reasons for accepting/declining trial participation and factors that influenced these decisions.
iii. Explore patients' experiences of trial participation, including perceived acceptability of being in the intervention/control arms and undergoing outcome assessments (e.g. CPET, wearing accelerometers).

We will address the above objectives by conducting semi-structured interviews with healthcare professionals and patients who accept and decline trial participation. Both healthcare professionals and patients will be sampled across all six sites. We anticipate up to 40 interviews will be conducted (20 patients, 20 clinical professionals) over the two-year study period.

Progression to full-scale RCT
This feasibility study aims to test the processes of a proposed future multicentre RCT and therefore we will utilise a traffic-light approach to determine trial progression according to the following indices:

- Patient participation:
  - Green: > 40% of approached patients consent to trial participation
  - Amber: Between 30% and 40% of approached patients consent
  - Red: < 30% of patients consent

- Adherence to randomisation and study completion:
  - Green: > 85% of enrolled participants received the allocated intervention and completed both CPETs without withdrawing

For peer review only

- Amber: Between 75% and 85% completion rate
- Red: < 75% completion rate

We anticipate that a green light for the above indices will realise patient recruitment and retention rates within a future full-scale RCT to enable scheduled completion. In response to an amber trigger, the Trial Management Group (TMG) will develop strategies that specifically respond to potential issues with patient participation that have been highlighted by the qualitative analysis. We would not proceed to a full-scale trial if a red signal is triggered.

During this feasibility study, we expect that, once open, each of the participating centres recruits a minimum of one patient per month and that fistula disconnection is performed within 62 days of CPET. If not met, the TMG will attempt to identify and address systematic problems that may hinder the main RCT.

Sample size
As this is a feasibility study, formal sample size calculation is not required. We will therefore aim to recruit forty patients (in keeping with recommendations (42)), twenty in each arm, with progression to a future RCT dependent on meeting set criteria relating to participant recruitment and retention rates as above. If the percentage of patients completing the study is 85%, 40 patients will give a 95% confidence interval of (70.2, 94.3).

Randomisation
Randomisation of consenting participants will be stratified according to fistula site (wrist or elbow) and fistula volume flow (low or high), as determined by a pre-randomisation Doppler ultrasound of the fistula, and allocated in a 1:1 ratio to the intervention (surgical disconnection of fistula) or to standard care (continued conservative management) using an interactive web response system provided by Sealed Envelope. Fistula flow rate will be measured during baseline assessment visits and categorised as either low flow (wrist AVF: ≤800 mL/min, elbow AVF: ≤1400 mL/min) or high flow (wrist AVF: >800 mL/min, elbow AVF: >1400 mL/min). The research team will confirm to the clinical and surgical teams which arm of the trial the participant has been randomised to, with the intervention being performed by a vascular access/transplant surgeon within 62 days of completion of baseline assessments. Given the surgical nature of the intervention, there will be no blinding in this trial with all participants, clinical and research team members aware of which trial arm is assigned at randomisation.

Statistical methods
The analysis of the primary outcome of the proportion of approached patients who complete the study successfully will include the proportion of eligible approached patients who consented, received the allocated intervention, completed both CPET tests and did not withdraw, with 95% confidence intervals. As part of the intention-to-treat approach, those who have graft failure, die, or are lost to follow-up will be included in the analysis of the primary outcome as not completing the study successfully. The component proportions and the corresponding 95% confidence intervals of the primary outcome will be presented as secondary outcomes.

The secondary feasibility outcomes of recruitment rate by trial site and the proportion of participants who were compliant with wearing wrist accelerometers, and their 95% confidence intervals, will also be presented. Compliance to wearing the monitor will be assessed by measuring wear time. The days from providing consent to first CPET and the days from CPET to fistula disconnection will be summarised, and the median and inter-quartile range presented. Patient and healthcare professional perceived acceptability of the trial design and processes will be analysed using qualitative analysis.

Descriptive statistics of the secondary clinical outcomes will be presented for each arm separately, at baseline, the six-month assessment, and the average change over time, with 95% confidence intervals. Peak VO_2 will also be presented by fistula site and according to pre-specified flow rate categories. Very limited hypothesis testing will be conducted but mixed linear regression will be used to examine any difference in peak VO_2 at six months between the two treatment arms, with adjustment for fistula site, flow rate, baseline peak VO_2 and centre. Fistula-related symptoms will be assessed using the modified VASQoL questionnaire for all participants at enrolment and at six months for the standard care arm and compared where applicable. The proportion of participants who experienced at least one MACE by six

For peer review only

months will be presented (alongside 95% confidence intervals), and details of all reported MACEs listed. In addition, summaries of all secondary safety outcomes will be presented by arm.

PATIENT AND PUBLIC INVOLVEMENT

The views of both kidney transplant patients and members of the public were sought during the design of this study, both through Patient and Public Advisory Group (PPAG) meetings and distributed surveys, with final oversight from local kidney patient groups. Key aspects of the study protocol were developed following these discussions with both adaptation to the study inclusion criteria and greater emphasis placed on the incorporation of QoL assessment and daily functioning.

The feasibility study has been designed such that continued patient involvement will be a core aspect, with the integrated qualitative research aimed at capturing the participant experience and generating insight into the factors that may undermine recruitment and threaten the viability of a full-scale RCT. We have included a patient representative as part of the TMG and will also conduct six-monthly PPI meetings during the feasibility study, with lay-person involvement, to moderate discussions with patient and clinicians on their views of trial participation.

We have already established close links with local kidney patient groups at each of the trial centres; the qualitative analysis will be shared with these groups; and we anticipate that the ensuing discussions with both patients and public will promote awareness of the study, facilitate patient recruitment and retention as well as support the dissemination of results and design of the subsequent RCT.

ETHICS AND DISSEMINATION

This study has been approved by the East Midlands – Derby Research Ethics Committee (22/EM/0002) and the Health Research Authority. Research will be conducted in compliance with the approved protocol, the Declaration of Helsinki (2013), the Principles of Good Clinical Practice, the UK Data Protection Act, the UK Framework for Health and Social Care Research and any other applicable national regulations. Written informed consent will be obtained from the participants by a suitably qualified member of the clinical/research team who has received trial training. The patient's right to refuse participation in the trial and withdraw from the study at any time and without reason will be respected, without prejudice to their further treatment.

The results of this trial will be presented to academic and non-academic groups with PPAG involvement in disseminating the study findings into the public domain. Open access, peer reviewed academic outputs and research reports together with associated summaries and key findings will be produced and held on the study website.

ACCESS TO DATA

Access to the final dataset will be available upon request from Prof Gavin Pettigrew, gjp25@cam.ac.uk, after the trial results have been published (expected September 2024) and should continue to be available for four years. No identifiable data will be shared. All participants have provided their written informed consent for their data to be used in this way.

DISCUSSION

There is no clear guideline on the management of AVFs following kidney transplantation. However, with the known burden of cardiovascular disease amongst renal transplant recipients, and the existing evidence that AVF disconnection improves cardiac structure, there is an increasing need to establish the role of AVF ligation in improving cardiovascular outcomes. Given the varying opinions of transplant patients and their clinicians on AVF preservation versus ligation, the COBALT feasibility study is a necessary requirement prior to pursuing a definitive robust RCT. With its embedded qualitative component, this feasibility trial aims to gauge the acceptability of the proposed trial design and optimise patient recruitment, with pre-defined stop-go criteria to justify progression to a full-scale RCT.

Acknowledgements

We thank Lewis Griffiths (MRC Epidemiology Unit) for their support for the physical activity measurement collection. We also give special thanks to Andrew Norton, our patient representative, who was instrumental in helping us develop this study proposal and securing support and funding from our local kidney patient association.

For peer review only

Collaborators

Subhankar Paul, Arturs Fedotov (Addenbrooke's Hospital, Cambridge); Mohammad Ayaz Hossain, John Tobi Ayorinde (Royal Free Hospital, London); Joshua Barnes (Royal Papworth Hospital, Cambridge); Carolyn Thomson, Olivia Steenson, Amy Cumming, Rowan McDougall, Nicola Thomson, Aileen Campbell, Steven Brown, Leigh Bainbridge, Louise McLeod, Kaye Travers, Socrates Stoumpos (Queen Elizabeth University Hospital, Glasgow); Helen Thomas, Alice Newton (NHS Blood and Transplant Clinical Trials Unit); Lucy Wallis (University of Bristol)

Author Contributions

VS, SK and GJP conceived the study. VS, EA, PBM, RM, JPH, AA, DS, KLR, LR, MG, KS, CH, JB, AS, AN, MS, MB, SK, GJP all contributed to the study design, refinement and implementation of the protocol and approved the manuscript. All members of the COBALT trial group were involved in the development of the study. SK and GJP are joint chief investigators.

Funding statement

This work was supported by National Institute for Health Research (NIHR) Research for Patient Benefit Funding grant (NIHR202255) with further support from the Addenbrooke's Kidney Patient Association and Cambridge University Hospitals NHS Foundation Trust. KLR is supported by the National Institute of Health Research (NIHR) Biomedical Research Centre in Cambridge (IS-BRC-1215-20014).

Competing interests statement

None declared.

For peer review only

REFERENCES

1. National Institute for Health and Care Excellence. Renal replacement therapy and conservative management - NICE guideline [NG107]. London: NICE; 2018.
2. RA W, VB A, EL M, AO O, RE E, LY A, et al. Comparison of mortality in all patients on dialysis, patients on dialysis awaiting transplantation, and recipients of a first cadaveric transplant. The New England journal of medicine. 1999;341(23).
3. A L, P K, N P, H K, B F, C W, et al. A study of the quality of life and cost-utility of renal transplantation. Kidney international. 1996;50(1).
4. CG R, KE T, JD R, DN C. Comparison of mortality risk for dialysis patients and cadaveric first renal transplant recipients in Ontario, Canada. Journal of the American Society of Nephrology : JASN. 2000;11(5).
5. UK Renal Registry. UK Renal Registry 21st Annual Report – data to 31/12/2017. Bristol, UK; 2019.
6. Karim A, Farrugia D, Cheshire J, Mahboob S, Begaj I, Ray D, et al. Recipient age and risk for mortality after kidney transplantation in England. Transplantation. 2014;97(8):832-8.
7. Kramer A, Boenink R, Noordzij M, Bosdriesz JR, Stel VS, Beltrán P, et al. The ERA-EDTA Registry Annual Report 2017: a summary. Clinical Kidney Journal. 2020.
8. Jardine AG, Gaston RS, Fellstrom BC, Holdaas H. Prevention of cardiovascular disease in adult recipients of kidney transplants. Lancet. 2011;378(9800):1419-27.
9. Arce CM, Lenihan CR, Montez-Rath ME, Winkelmayer WC. Comparison of longer-term outcomes after kidney transplantation between Hispanic and non-Hispanic whites in the United States. Am J Transplant. 2015;15(2):499-507.
10. Domenick Sridharan N, Fish L, Yu L, Weisbord S, Jhamb M, Makaroun MS, et al. The associations of hemodialysis access type and access satisfaction with health-related quality of life. J Vasc Surg. 2018;67(1):229-35.
11. Korevaar JC, Merkus MP, Jansen MA, Dekker FW, Boeschoten EW, Krediet RT, et al. Validation of the KDQOL-SF: a dialysis-targeted health measure. Qual Life Res. 2002;11(5):437-47.
12. Pastan S, Soucie JM, McClellan WM. Vascular access and increased risk of death among hemodialysis patients. Kidney Int. 2002;62(2):620-6.
13. Polkinghorne KR, McDonald SP, Atkins RC, Kerr PG. Vascular access and all-cause mortality: a propensity score analysis. J Am Soc Nephrol. 2004;15(2):477-86.
14. Kumwenda M, Mitra S, Reid C. Vascular access for haemodialysis. UK Renal Association; 2015.
15. Aitken E, Kerr D, Geddes C, Berry C, Kingsmore D. Cardiovascular changes occurring with occlusion of a mature arteriovenous fistula. J Vasc Access. 2015;16(6):459-66.
16. Hartley JL, Sharma A, Taha L, Hestletine T. High-output cardiac failure secondary to high-output arteriovenous fistula: investigations, management and definitive treatment. BMJ Case Rep. 2020;13(2).
17. Wasse H, Singapuri MS. High-output heart failure: how to define it, when to treat it, and how to treat it. Semin Nephrol. 2012;32(6):551-7.
18. MacRae JM, Pandeya S, Humen DP, Krivitski N, Lindsay RM. Arteriovenous fistula-associated high-output cardiac failure: a review of mechanisms. Am J Kidney Dis. 2004;43(5):e17-22.
19. Levy D, Garrison RJ, Savage DD, Kannel WB, Castelli WP. Prognostic implications of echocardiographically determined left ventricular mass in the Framingham Heart Study. N Engl J Med. 1990;322(22):1561-6.
20. Tsao CW, Gona PN, Salton CJ, Chuang ML, Levy D, Manning WJ, et al. Left Ventricular Structure and Risk of Cardiovascular Events: A Framingham Heart Study Cardiac Magnetic Resonance Study. J Am Heart Assoc. 2015;4(9):e002188.
21. Zheng H, Bu S, Song Y, Wang M, Wu J, Chen J. To Ligate or Not to Ligate: A Meta-analysis of Cardiac Effects and Allograft Function following Arteriovenous Fistula Closure in Renal Transplant Recipients. Ann Vasc Surg. 2020;63:287-92.
22. Rao NN, Stokes MB, Rajwani A, Ullah S, Williams K, King D, et al. Effects of Arteriovenous Fistula Ligation on Cardiac Structure and Function in Kidney Transplant Recipients. Circulation. 2019;139(25):2809-18.
23. Society AT, Physicians ACoC. ATS/ACCP Statement on cardiopulmonary exercise testing. Am J Respir Crit Care Med. 2003;167(2):211-77.
24. Myers J, Prakash M, Froelicher V, Do D, Partington S, Atwood JE. Exercise capacity and mortality among men referred for exercise testing. N Engl J Med. 2002;346(11):793-801.

For peer review only

25. Kodama S, Saito K, Tanaka S, Maki M, Yachi Y, Asumi M, et al. Cardiorespiratory fitness as a quantitative predictor of all-cause mortality and cardiovascular events in healthy men and women: a meta-analysis. JAMA. 2009;301(19):2024-35.
26. Swank AM, Horton J, Fleg JL, Fonarow GC, Keteyian S, Goldberg L, et al. Modest increase in peak VO2 is related to better clinical outcomes in chronic heart failure patients: results from heart failure and a controlled trial to investigate outcomes of exercise training. Circ Heart Fail. 2012;5(5):579-85.
27. Ting SM, Iqbal H, Kanji H, Hamborg T, Aldridge N, Krishnan N, et al. Functional cardiovascular reserve predicts survival pre-kidney and post-kidney transplantation. J Am Soc Nephrol. 2014;25(1):187-95.
28. Sietsema KE, Amato A, Adler SG, Brass EP. Exercise capacity as a predictor of survival among ambulatory patients with end-stage renal disease. Kidney Int. 2004;65(2):719-24.
29. Ross R, Blair SN, Arena R, Church TS, Després JP, Franklin BA, et al. Importance of Assessing Cardiorespiratory Fitness in Clinical Practice: A Case for Fitness as a Clinical Vital Sign: A Scientific Statement From the American Heart Association. Circulation. 2016;134(24):e653-e99.
30. Imboden MT, Harber MP, Whaley MH, Finch WH, Bishop DL, Fleenor BS, et al. The Association between the Change in Directly Measured Cardiorespiratory Fitness across Time and Mortality Risk. Prog Cardiovasc Dis. 2019;62(2):157-62.
31. Hager A, Hess J. Comparison of health related quality of life with cardiopulmonary exercise testing in adolescents and adults with congenital heart disease. Heart. 2005;91(4):517-20.
32. Athanasopoulos LV, Dritsas A, Doll HA, Cokkinos DV. Explanation of the variance in quality of life and activity capacity of patients with heart failure by laboratory data. Eur J Cardiovasc Prev Rehabil. 2010;17(4):375-9.
33. Huff CM, Turer AT, Wang A. Correlations between physician-perceived functional status, patient-perceived health status, and cardiopulmonary exercise results in hypertrophic cardiomyopathy. Qual Life Res. 2013;22(3):647-52.
34. Eriksen L, Curtis T, Grønbæk M, Helge JW, Tolstrup JS. The association between physical activity, cardiorespiratory fitness and self-rated health. Prev Med. 2013;57(6):900-2.
35. Edelmann F, Gelbrich G, Düngen HD, Fröhling S, Wachter R, Stahrenberg R, et al. Exercise training improves exercise capacity and diastolic function in patients with heart failure with preserved ejection fraction: results of the Ex-DHF (Exercise training in Diastolic Heart Failure) pilot study. J Am Coll Cardiol. 2011;58(17):1780-91.
36. Kitzman DW, Brubaker PH, Herrington DM, Morgan TM, Stewart KP, Hundley WG, et al. Effect of endurance exercise training on endothelial function and arterial stiffness in older patients with heart failure and preserved ejection fraction: a randomized, controlled, single-blind trial. J Am Coll Cardiol. 2013;62(7):584-92.
37. Delanaye P, Mariat C. The applicability of eGFR equations to different populations. Nat Rev Nephrol. 2013;9(9):513-22.
38. Shield CF, McGrath MM, Goss TF. Assessment of health-related quality of life in kidney transplant patients receiving tacrolimus (FK506)-based versus cyclosporine-based immunosuppression. FK506 Kidney Transplant Study Group. Transplantation. 1997;64(12):1738-43.
39. Richarz S, Greenwood S, Kingsmore DB, Thomson PC, Dunlop M, Bouamrane MM, et al. Validation of a vascular access specific quality of life measure (VASQoL). J Vasc Access. 2021:11297298211046746.
40. Holdaas H, Fellström B, Jardine AG, Holme I, Nyberg G, Fauchald P, et al. Effect of fluvastatin on cardiac outcomes in renal transplant recipients: a multicentre, randomised, placebo-controlled trial. Lancet. 2003;361(9374):2024-31.
41. Dindo D, Demartines N, Clavien PA. Classification of surgical complications: a new proposal with evaluation in a cohort of 6336 patients and results of a survey. Ann Surg. 2004;240(2):205-13.
42. Lancaster GA, Dodd S, Williamson PR. Design and analysis of pilot studies: recommendations for good practice. J Eval Clin Pract. 2004;10(2):307-12.

For peer review only

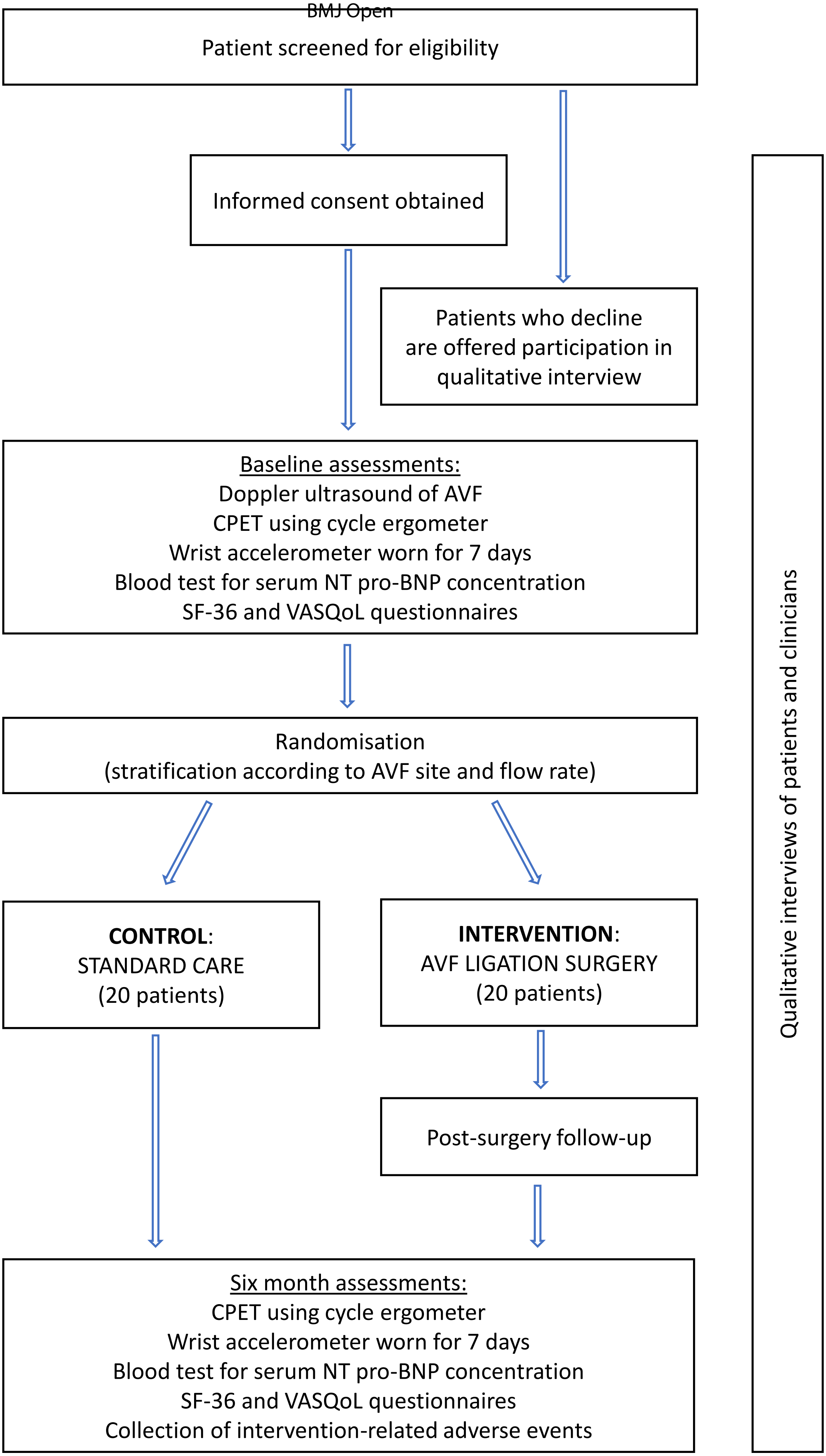

For peer review only - http://bmjopen.bmj.com/site/about/guidelines.xhtml

Figure 1: Overall design of COBALT study. AVF, Arteriovenous fistula CPET, Cardiopulmonary exercise test; NT pro-BNP, N-terminal pro-B-type natriuretic peptide; SF-36, Short Form 36; VASQoL, Vascular Access Specific Quality of Life

COBALT

Participant Identification Number: _______________

University of BRISTOL

TRUST LOGO

Informed Consent Form for Healthcare Professionals

Should we ligate haemodialysis fistulas in patients after they have been transplanted successfully: the **COBALT** feasibility study (**C**ardiorespiratory **O**ptimisation **B**y **A**VF **L**igation after **T**ransplantation).

Please initial the boxes if you agree with the following statements:

1. I confirm that I have read the Healthcare Professionals information sheet dated.................. (version...........) for the above study. I have had the opportunity to consider the information, ask questions and have had these answered satisfactorily. ☐
2. I agree to taking part in an audio-recorded interview. ☐
3. I understand that I am free to withdraw from the interview study at any time, without giving a reason, and that withdrawing will not affect my legal rights. I understand that withdrawal of data already provided will not be possible after 2 weeks of recordings having taken place. ☐
4. I agree to take part in the above study. ☐

Optional statements (please initial either the yes or no box):

Statement	Yes	No
5. I am happy to be contacted in the future for a potential follow-up interview, and understand I have a right to accept/decline taking part at the time.	☐	☐
6. I agree to my audio-recorded interviews being transferred to and retained by the University of Bristol and their authorised representatives for transcription, training, teaching and research purposes, now and in the future.	☐	☐
7. I agree to anonymised data from my interviews being made "Controlled access" after the study, and understand this means data will be stored indefinitely and may be used for purposes not related to this study, although it will not be possible to identify me from these data.	☐	☐

Name of Participant	Date	Signature
Name of person taking consent	Date	Signature

When completed: 1 for participant; 1 for researcher

For peer review only - http://bmjopen.bmj.com/site/about/guidelines.xhtml

COBALT

Insert Trust logo

IRAS ID: 305610

Title of Study: Cardiorespiratory Optimisation By Arteriovenous fistula Ligation after Transplantation (COBALT)

Centre Number: ___________

Name of Researcher: ______________________________

Participant Identification Number for this study: __________________

For peer review only

INFORMED CONSENT FORM

Please initial the boxes if you agree with the following statements:

1. I confirm that I have read the information sheet dated................... (version........) for the above study and that the trial procedures and information have been explained to me. I have had the opportunity to consider the information, ask questions and have had these answered satisfactorily. ☐

2. I understand that my participation is voluntary and that I am free to withdraw at any time without giving any reason, without my medical care or legal rights being affected. ☐

3. I understand that relevant sections of my medical notes and data collected during the study, may be looked at by individuals from Cambridge University Hospitals NHS Foundation Trust / University of Cambridge / NHS Blood and Transplant, from regulatory authorities or from the NHS Trust, where it is relevant to my taking part in this research. I give permission for these individuals to have access to my records. ☐

4. I understand that the information collected about me will be used to support other ethically approved research in the future, and may be shared anonymously with other researchers in the UK or abroad. ☐

For peer review only - http://bmjopen.bmj.com/site/about/guidelines.xhtml

COBALT

Insert Trust logo

5. I agree to take part in the COBALT study. ☐

Optional statements (please initial either the yes or no box): **Yes** **No**

6. I agree to my General Practitioner and Nephrologist (kidney doctor) being informed of my participation in the COBALT study. ☐ ☐

7. I agree that my name and contact details can be securely shared with researchers at the University of Bristol and that those researchers can contact me to arrange an interview about my experience of being approached to take part in the COBALT study (and, my experiences of participation, if relevant). ☐ ☐

8. I agree to anonymised data from my interviews being made “Controlled access” after the study and understand this means data will be stored indefinitely and may be used for purposes not related to this study, although it will not be possible to identify me from these data. ☐ ☐

Name of Participant	Date	Signature
Name of Researcher receiving consent	Date	Signature

1 x copy for participant
1 x copy for COBALT file to be kept at hospital site
1 x copy for participant’s hospital medical record

For peer review only - http://bmjopen.bmj.com/site/about/guidelines.xhtml

Reporting checklist for protocol of a clinical trial.

Based on the SPIRIT guidelines.

Instructions to authors

Complete this checklist by entering the page numbers from your manuscript where readers will find each of the items listed below.

Your article may not currently address all the items on the checklist. Please modify your text to include the missing information. If you are certain that an item does not apply, please write "n/a" and provide a short explanation.

Upload your completed checklist as an extra file when you submit to a journal.

In your methods section, say that you used the SPIRITreporting guidelines, and cite them as:

Chan A-W, Tetzlaff JM, Gøtzsche PC, Altman DG, Mann H, Berlin J, Dickersin K, Hróbjartsson A, Schulz KF, Parulekar WR, Krleža-Jerić K, Laupacis A, Moher D. SPIRIT 2013 Explanation and Elaboration: Guidance for protocols of clinical trials. BMJ. 2013;346:e7586

		Reporting Item	Page Number
**Administrative information**			
Title	#1	Descriptive title identifying the study design, population, interventions, and, if applicable, trial acronym	2

For peer review only

Trial registration	#2a	Trial identifier and registry name. If not yet registered, name of intended registry	4
Trial registration: data set	#2b	All items from the World Health Organization Trial Registration Data Set	4
Protocol version	#3	Date and version identifier	4
Funding	#4	Sources and types of financial, material, and other support	12
Roles and responsibilities: contributorship	#5a	Names, affiliations, and roles of protocol contributors	2
Roles and responsibilities: sponsor contact information	#5b	Name and contact information for the trial sponsor	4
Roles and responsibilities: sponsor and funder	#5c	Role of study sponsor and funders, if any, in study design; collection, management, analysis, and interpretation of data; writing of the report; and the decision to submit the report for publication, including whether they will have ultimate authority over any of these activities	n/a
Roles and responsibilities: committees	#5d	Composition, roles, and responsibilities of the coordinating centre, steering committee, endpoint adjudication committee, data management team, and	n/a

For peer review only

		other individuals or groups overseeing the trial, if applicable (see Item 21a for data monitoring committee)	
**Introduction**			
Background and rationale	#6a	Description of research question and justification for undertaking the trial, including summary of relevant studies (published and unpublished) examining benefits and harms for each intervention	6
Background and rationale: choice of comparators	#6b	Explanation for choice of comparators	6
Objectives	#7	Specific objectives or hypotheses	7
Trial design	#8	Description of trial design including type of trial (eg, parallel group, crossover, factorial, single group), allocation ratio, and framework (eg, superiority, equivalence, non-inferiority, exploratory)	7
**Methods: Participants, interventions, and outcomes**			
Study setting	#9	Description of study settings (eg, community clinic, academic hospital) and list of countries where data will be collected. Reference to where list of study sites can be obtained	7

For peer review only

Eligibility criteria	#10	Inclusion and exclusion criteria for participants. If applicable, eligibility criteria for study centres and individuals who will perform the interventions (eg, surgeons, psychotherapists)	7
Interventions: description	#11a	Interventions for each group with sufficient detail to allow replication, including how and when they will be administered	8
Interventions: modifications	#11b	Criteria for discontinuing or modifying allocated interventions for a given trial participant (eg, drug dose change in response to harms, participant request, or improving / worsening disease)	n/a
Interventions: adherance	#11c	Strategies to improve adherence to intervention protocols, and any procedures for monitoring adherence (eg, drug tablet return; laboratory tests)	n/a
Interventions: concomitant care	#11d	Relevant concomitant care and interventions that are permitted or prohibited during the trial	n/a
Outcomes	#12	Primary, secondary, and other outcomes, including the specific measurement variable (eg, systolic blood pressure), analysis metric (eg, change from baseline, final value, time to event), method of aggregation (eg, median, proportion), and time point for each outcome. Explanation of the clinical relevance of chosen efficacy and harm outcomes is strongly recommended	8

For peer review only

Participant timeline	#13	Time schedule of enrolment, interventions (including any run-ins and washouts), assessments, and visits for participants. A schematic diagram is highly recommended (see Figure)	See Figure
Sample size	#14	Estimated number of participants needed to achieve study objectives and how it was determined, including clinical and statistical assumptions supporting any sample size calculations	9
Recruitment	#15	Strategies for achieving adequate participant enrolment to reach target sample size	7
**Methods: Assignment of interventions (for controlled trials)**			
Allocation: sequence generation	#16a	Method of generating the allocation sequence (eg, computer-generated random numbers), and list of any factors for stratification. To reduce predictability of a random sequence, details of any planned restriction (eg, blocking) should be provided in a separate document that is unavailable to those who enrol participants or assign interventions	10
Allocation concealment mechanism	#16b	Mechanism of implementing the allocation sequence (eg, central telephone; sequentially numbered, opaque,	10

For peer review only

		sealed envelopes), describing any steps to conceal the sequence until interventions are assigned	
Allocation: implementation	#16c	Who will generate the allocation sequence, who will enrol participants, and who will assign participants to interventions	10
Blinding (masking)	#17a	Who will be blinded after assignment to interventions (eg, trial participants, care providers, outcome assessors, data analysts), and how	n/a
Blinding (masking): emergency unblinding	#17b	If blinded, circumstances under which unblinding is permissible, and procedure for revealing a participant's allocated intervention during the trial	n/a
**Methods: Data collection, management, and analysis**			
Data collection plan	#18a	Plans for assessment and collection of outcome, baseline, and other trial data, including any related processes to promote data quality (eg, duplicate measurements, training of assessors) and a description of study instruments (eg, questionnaires, laboratory tests) along with their reliability and validity, if known. Reference to where data collection forms can be found, if not in the protocol	8

For peer review only

Data collection plan: retention	#18b	Plans to promote participant retention and complete follow-up, including list of any outcome data to be collected for participants who discontinue or deviate from intervention protocols	n/a
Data management	#19	Plans for data entry, coding, security, and storage, including any related processes to promote data quality (eg, double data entry; range checks for data values). Reference to where details of data management procedures can be found, if not in the protocol	n/a
Statistics: outcomes	#20a	Statistical methods for analysing primary and secondary outcomes. Reference to where other details of the statistical analysis plan can be found, if not in the protocol	8
Statistics: additional analyses	#20b	Methods for any additional analyses (eg, subgroup and adjusted analyses)	n/a
Statistics: analysis population and missing data	#20c	Definition of analysis population relating to protocol non-adherence (eg, as randomised analysis), and any statistical methods to handle missing data (eg, multiple imputation)	n/a

Methods: Monitoring

Data monitoring: formal committee	#21a	Composition of data monitoring committee (DMC); summary of its role and reporting structure; statement of whether it is independent from the sponsor and competing interests; and reference to where further	n/a

For peer review only

		details about its charter can be found, if not in the protocol. Alternatively, an explanation of why a DMC is not needed	
Data monitoring: interim analysis	#21b	Description of any interim analyses and stopping guidelines, including who will have access to these interim results and make the final decision to terminate the trial	n/a
Harms	#22	Plans for collecting, assessing, reporting, and managing solicited and spontaneously reported adverse events and other unintended effects of trial interventions or trial conduct	n/a
Auditing	#23	Frequency and procedures for auditing trial conduct, if any, and whether the process will be independent from investigators and the sponsor	n/a
**Ethics and dissemination**			
Research ethics approval	#24	Plans for seeking research ethics committee / institutional review board (REC / IRB) approval	11
Protocol amendments	#25	Plans for communicating important protocol modifications (eg, changes to eligibility criteria, outcomes, analyses) to relevant parties (eg, investigators, REC / IRBs, trial participants, trial registries, journals, regulators)	n/a

For peer review only

Consent or assent	#26a	Who will obtain informed consent or assent from potential trial participants or authorised surrogates, and how (see Item 32)	7
Consent or assent: ancillary studies	#26b	Additional consent provisions for collection and use of participant data and biological specimens in ancillary studies, if applicable	n/a
Confidentiality	#27	How personal information about potential and enrolled participants will be collected, shared, and maintained in order to protect confidentiality before, during, and after the trial	n/a
Declaration of interests	#28	Financial and other competing interests for principal investigators for the overall trial and each study site	12
Data access	#29	Statement of who will have access to the final trial dataset, and disclosure of contractual agreements that limit such access for investigators	11
Ancillary and post trial care	#30	Provisions, if any, for ancillary and post-trial care, and for compensation to those who suffer harm from trial participation	n/a
Dissemination policy: trial results	#31a	Plans for investigators and sponsor to communicate trial results to participants, healthcare professionals, the public, and other relevant groups (eg, via publication, reporting in results databases, or other data sharing arrangements), including any publication restrictions	11

For peer review only

Dissemination policy: authorship	#31b	Authorship eligibility guidelines and any intended use of professional writers	12
Dissemination policy: reproducible research	#31c	Plans, if any, for granting public access to the full protocol, participant-level dataset, and statistical code	11

Appendices

Informed consent materials	#32	Model consent form and other related documentation given to participants and authorised surrogates	n/a
Biological specimens	#33	Plans for collection, laboratory evaluation, and storage of biological specimens for genetic or molecular analysis in the current trial and for future use in ancillary studies, if applicable	n/a

The SPIRIT Explanation and Elaboration paper is distributed under the terms of the Creative Commons Attribution License CC-BY-NC. This checklist was completed on 20. August 2022 using https://www.goodreports.org/, a tool made by the EQUATOR Network in collaboration with Penelope.ai

For peer review only
